# Development of a host-signature-based machine learning model to diagnose bacterial and viral infections in febrile children

**DOI:** 10.3389/fped.2025.1608812

**Published:** 2025-08-06

**Authors:** Fang Bai, Zelong Gong, Dong Cui, Xiaomei Zhang, Wenteng Hong, Yi Gao, Kai Lin, Weijie Chen, Lu Li, Juan Huang, Biying Zheng, Junfa Xu, Na Xiao

**Affiliations:** ^1^ Dongguan Key Laboratory of Pathogenesis and Experimental Diagnosis of Infectious Diseases, Institute of Laboratory Medicine of School of Medical Technology, The First Dongguan Affiliated Hospital, Guangdong Medical University, Dongguan, Guangdong, China; ^2^ Yantian District Center for Disease Control and Prevention (CDC), Shenzhen, Guangdong, China

**Keywords:** machine learning, bacteria, virus, febrile children, host gene signatures, diagnosis

## Abstract

**Background:**

Early aetiological diagnosis is critical for the management of febrile children with infectious illness, as it strongly influences the choice of appropriate medication and can affect a child's complications and outcome. New diagnostic strategies based on host genes have recently been developed and have achieved high accuracy and clinical practicability. In this study, through integrative bioinformatics analysis, we aimed to construct artificial neural network (ANN, multilayer perceptron) and random forest (RF) models based on host gene signatures to diagnose bacterial or viral (B/V) infection in febrile children.

**Results:**

Transcriptome data from the whole blood of children were collected from a public database. Of these, 384 febrile young children (definite bacterial: *n* = 135, definite viral: *n* = 249) were involved in the construction of the RF model. For the generalized RF model, 1,042 patients were included with various aetiological infections, such as *Staphylococcus aureus*, pathogenic *Escherichia coli*, *Salmonella*, *Shigella*, *adenovirus*, *HHV6*, *enterovirus, rhinovirus, human rotavirus, human norovirus*, and *influenza A pneumonia*. The overlap of 57 candidate genes between the 117 differentially expressed genes (DEGs) and the 264 module member genes was identified through DEGs analysis and weighted gene co-expression network analysis (WGCNA). Subsequently, L1 regularization algorithms and variable significance analysis (multilayer perceptron) were used to simplify and rank the predictive features, and LCN2 (100.0%), IFI27 (84.4%), SLPI (63.2%), IFIT2 (44.6%) and PI3 (44.5%) were identified as the top predictors. By utilizing the transformed value RefValue (i) of these five genes, the RF model achieved an AUC of 0.9917 in training and 0.9517 in testing for diagnosing B/V infection in children. The ANN model achieved an AUC of 0.9540 in testing. Furthermore, a generalized RF model involving 1,042 patients was developed to predict different aetiological types of samples, achieving an AUC of 0.9421 in training and 0.8968 in testing.

**Conclusions:**

A five-gene host signature (IFIT2, SLPI, IFI27, LCN2, and PI3) was identified and successfully used to construct an RF model that distinguishes B/V infection in febrile children, achieving 85.3% accuracy, 95.1% sensitivity, and 80.0% specificity, and to construct an ANN model that achieves 92.4% accuracy, 86.8% sensitivity, and 95% specificity.

## 1 Introduction

The severity of febrile illnesses is commonly underestimated because of their diverse aetiologies, which include infectious diseases, autoimmune responses, and other causes (1–3). Annually, millions of people around the world, particularly children, are affected by infectious diseases, which may result in permanent disabilities or fatal outcomes (4). Empirical drug therapy can increase the occurrence of drug resistance and the risk of adverse side effects (5, 6). Hence, early aetiological diagnosis for bacterial or viral (B/V) infection is critical for the management of febrile children with infectious illness, as it greatly influences the selection of appropriate medication (7). Intrinsically, infectious disease diagnosis relies on the strategy of aetiological hypotheses followed by corresponding validation. The traditional pathogen culture method is time-consuming, has low sensitivity and requires significant experience in microbiology (8, 9). On the other hand, nucleic acid and serological testing require accurate aetiological hypotheses to guide the direction of testing.

Conventional biomarkers for the initial assessment of bacterial or viral (B/V) infections include white blood cells (WBCs), lymphocytes (LYMs), C-reactive protein (CRP) and procalcitonin (PCT) (10–12). Nevertheless, methods to detect these infection-related markers face challenges in meeting clinical requirements because of their limited sensitivity and specificity (13). Recently, host gene signatures have been developed for rapid B/V diagnostics by detecting several host genes in whole blood samples, which may provide initial identification quickly (13–16). Host gene signatures detection is a novel diagnostic approach that focuses on changes in the host gene expression profile and differs from traditional pathogen-based detection methods (17). Currently, the exploration of host gene signatures for the diagnostic identification of febrile children with B/V infections is still in the early and imperfect stages. With the development of integrated bioinformatics analysis and machine learning algorithms, there is considerable potential for improving host gene signatures detection in terms of practicality and generalizability.

With the ability to manage multiple datasets and analyse nonlinear relationships among a large number of features (18, 19), the random forest (RF) model has an advantage in handling the binary classification challenge of distinguishing between B/V infections in febrile children. In this study, a regularization algorithm (LASSO), artificial neural network (ANN), variable significance analysis (multilayer perceptron, MLP) and RF construction were integrated to improve the prediction of B/V infection. Moreover, intersection analysis was conducted on differentially expressed genes and co-expressed module genes through differentially expressed genes (DEGs) analysis and weighted gene co-expression network analysis (WGCNA) to obtain representative candidate biomarkers. The aim of our study is to identify host gene signatures and develop a practical machine learning model for diagnosing B/V infection in febrile children, which will guide decision-making regarding antibiotics or antiviral treatments in febrile children with an unknown infection type.

## 2 Methods

### 2.1 Data collection and preprocessing

Expression datasets of whole blood from febrile children with B/V infection were downloaded from the Gene Expression Omnibus (GEO) database (https://www.ncbi.nlm.nih.gov/gds/?term=). The database was screened using the following terms: (“childhood” OR “children”) AND (“bacterial” AND “viral”). Each dataset underwent individual assessment on the basis of the following criteria to determine inclusion in our analysis: data completeness (data completeness and availability), information concordance and whole-blood samples, etc. In this study, datasets GSE40396, GSE72809, GSE72810, and GSE73464 (comprising 384 samples) were ultimately selected for constructing the child RF prediction model. Datasets GSE40396, GSE72809, GSE72810, GSE73464, GSE40012, GSE69529, GSE63990, GSE42026, and GSE60244 (comprising 1,042 samples) were selected for building the generalized RF model (encompassing both children and adults). Basic information for the B/V infection datasets is shown in Table 1; Supplementary File 1.

**Table 1:** Overview of basic information for B/V infection datasets.

Accession number	Beadchip	Hospital	Febrile condition	Sample size		Reference
				Bacterial group	Viral group	
GSE72809	Illumina HumanHT-12 V4.0 expression beadchip	UK hospitals; GENDRES network, Santiago de Compostela); Rady Children's Hospital, San Diego	Axillary temperature ≥38°C	*n* = 52	*n* = 92	(15)
GSE72810	Illumina HumanHT-12 V3.0 expression beadchip	UK hospitals; GENDRES network, Santiago de Compostela); Rady Children's Hospital, San Diego	Axillary temperature ≥38°C	*n* = 23	*n* = 28	(15)
GSE40396	Illumina HumanHT-12 V4.0 expression beadchip	St. Louis Children's Hospital, USA	Temperature of 38°C or greater	*n* = 8	*n* = 35	(20, 21)
GSE73464	Illumina HumanHT-12 V4.0 expression beadchip	No mentioning directly	Febrile condition (not specified)	*n* = 52	*n* = 94	(22)
GSE40012	Illumina HumanHT-12 V3.0 expression beadchip	No mentioning directly	Temperature > 100.4°F (38°C)	*n* = 61	*n* = 39	(23)
GSE69529	Illumina HiSeq 2500 (Homo sapiens)	Hospital General O'Horan, Mexico	No mentioning	*n* = 123	*n* = 56	(24)
GSE63990	Affymetrix Human Genome U133A 2.0 Array	Emergency Departments of Duke University Medical Center, USA	Temperature <36°C or >38°C	*n* = 67	*n* = 41	(25)
GSE42026	Illumina HumanHT-12 V3.0 expression beadchip	Medical Center (DVAMC; Durham, NC), Henry Ford Hospital, USA	Febrile condition (not specified)	*n* = 18	*n* = 56	(14)
GSE60244	Illumina HumanHT-12 V3.0 expression beadchip	St Mary's Hospital, UK	Axillary temperature ≥38°C	*n* = 22	*n* = 71	(26)

The diagnostic criteria for the bacterial group and viral group in the involved datasets were as follows: (1) GSE72809-GSE72810: Bacterial group: confirmed by positive bacterial culture from a sterile site (e.g., blood, CSF), regardless of viral codetection. Viral group: confirmed by positive viral culture by molecular (PCR) or immunofluorescence testing, with no clinical/microbiological evidence of bacterial coinfection. (2) GSE40396: Bacterial Group: patients who had a definite bacterial infection (bacteremia, urinary tract infection, etc.) on the basis of positive bacterial culture. Viral Group: patients were considered positive if the indicated virus was detected in either the blood or the nasopharyngeal sample via virus-specific PCR. (3) GSE73464: Bacterial group: Bacterial cultures included blood, CSF, urine, and tissue. Pneumococcal antigen was tested in blood and urine; meningococcal and pneumococcal DNA were detected by PCR. Viral group: viral diagnostics of nasopharyngeal aspirates were performed by immunofluorescence (RSV, adenovirus, parainfluenza, influenza A/B) and nested PCR (expanded respiratory virus panel). Patients were categorized into disease groups after evaluation by 2 independent clinicians. The remaining datasets (GSE40012, GSE69529, GSE63990, GSE42026, and GSE60244) are described in detail in Table 1.

### 2.2 Intersecting DEGs analysis of multi-dataset

For analyzing and visualizing the transcriptomic data from public database, necessary R (version 4.4.1), *Bioconductor* packages *limma*, *DESeq2* and *ggplot* were applied in the environment. TBtools-II v2.119 software was applied to generate heatmap. *Bioinformatics & Evolutionary Genomics* online software (http://bioinformatics.psb.ugent.be/webtools/Venn/) was used for Venn diagram analysis. Intersecting DEGs are those that exhibit statistically significant differences when identified in more than three microarrays (see Supplementary Files 2, 3 for details).

### 2.3 WGCNA analysis

Following by data preparation, sample clustering, soft threshold selection, co-expression network construction and module membership analysis, the positively or negatively related modules and the related genes could be obtained for the subsequent analysis (see Supplementary File 3 for details).

### 2.4 Obtaining the candidate genes

*Bioinformatics & Evolutionary Genomics* was employed to generate the intersecting genes of DEGs and WGCNA output results. LASSO was conducted to reduce the variables by penalizing the regression coefficients with L1 penalty. Kyoto Encyclopedia of Genes and Genomes (KEGG) and Gene ontology (GO) were conducted by using R. Protein-protein interaction (PPI) network was constructed through the STRING database online tool (see Supplementary File 3 for details).

### 2.5 Immune infiltration analysis

*CIBERSORTx* provides 22 human immune cell types proportions through the input of gene expression profiling (https://cibersortx.stanford.edu/) (27, 28). Analysis Module: Input Cell Fractions, Signature matrix file: LM22 (22 immune cell type), Disable quantile normalization, Permutations for significance analysis: 100 permutations (see Supplementary File 3 for details).

### 2.6 ANN construction

Reference gene selection: Genes geomean of ranking values were calculated among candidate gene through *RefFinder*, which summarized the comprehensive stability of housekeeping gene among *Delta CT*, *BestKeeper*, *Normfinder* and *Genorm* (29). Data preprocessing: To enhance the predictive model extrapolation capability, mathematical preprocessing formula was utilized to decreasing data variability from various matrixes: RefValue(i)=Sigmoid[expr.value(i)/]expr.value (housekeepinggene). ANN (Multilayer perceptron) was analyzed and constructed by SPSS Statistics 20.0: diagnosis status (B/V infections) labels as dependent variable, RefValue (i) labels as covariate or factor. artificial neural networks (Multilayer perceptron) were analyzed and constructed by SPSS Statistics 20.0 (Training case/testing case = 7:3). According to *IBM SPSS Statistics Algorithms Manual*, cases are assigned to training or testing sets by generating a uniform random number for each case, training case/testing case = 7:3; number of units in hidden layer (2 layers), activation function in hidden layers: Hyperbolic tangent; Activation function in output layer: Softmax; Error function in output layer: Cross-entropy.

### 2.7 RF-based machine learning classification

Our study employs *tidymodels*, *rmda*, *fastshap*, *ggplot2*, *ggbeeswarm,* and *ggExtra* packages among others, for random forest-based machine learning classification. R was used for splitting the input data (traindata: testdata = 7:3). This step involves randomly shuffling the data and splitting it into test and validation sets to ensure there are no overlapping samples between them. As the sample size increases in RF generalized model, appropriately raising the proportion of training data can enhance the model's predictive performance. Therefore, we adjusted the input data split ratio to training data: test data = 7.5: 2.5. Data preprocessing, hyperparameter tuning with grid search or Bayesian optimization, model training, evaluation and SHapley additive explanation (SHAP) analysis were performed during the model building process. Number of variables (outcome: 1, predictor: 5 numeric variables); Parameter setting range: mtry [range = c (2, 10)], trees [range = c (100, 1,000)], min_n [range = c (7, 55)]. Prediction probability plot, receiver operating characteristic (ROC) plot, precision-recall plot, calibration plot, confusion matrix, Kolmogorov–Smirnov (KS) Plot and among others were generated by R tools.

### 2.8 Statistical analysis

Data processing and statistical evaluations were carried out utilizing R software (version 4.4.1), R studio (version 2024.4.2.764) and IBM SPSS Statistics (version 20.0). Partial graphs were conducted using *GraphPad Prism 9*, version 9.5.0 (730). The quantitative data are expressed as mea*n* ± standard deviation (SD). Two-group significances were analyzed by unpaired Student's *t* test. *P* *<* *0.05* was defined as statistically significant, *p *<* *0.05*, **p *<* *0.01*, ***p *<* *0.001*, ns, no significant.

## 3 Results

### 3.1 Identification of candidate DEGs with predictive value for B/V infection

**Figure 1:**
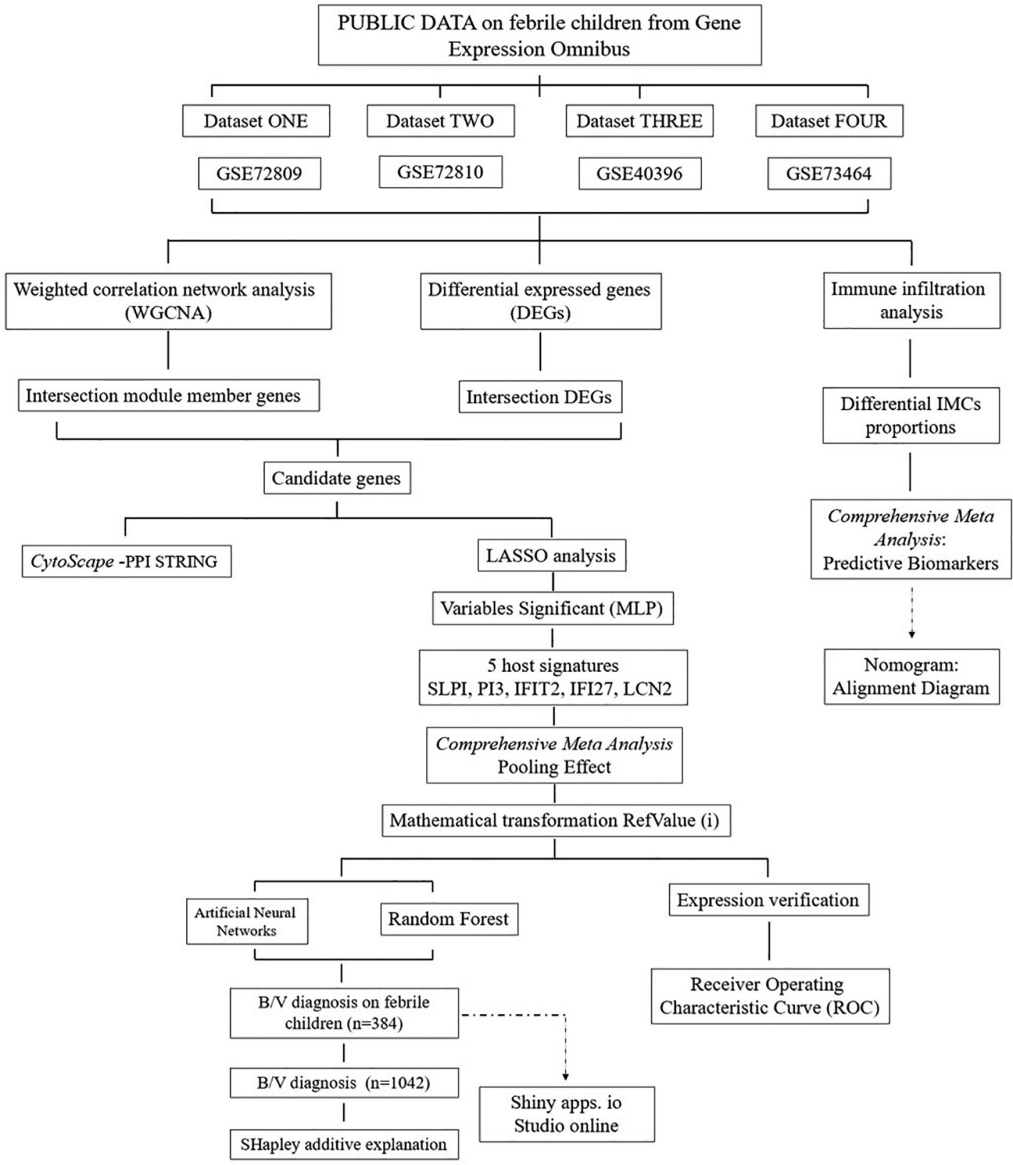
The systematic methodology of the study.

**Figure 2:**
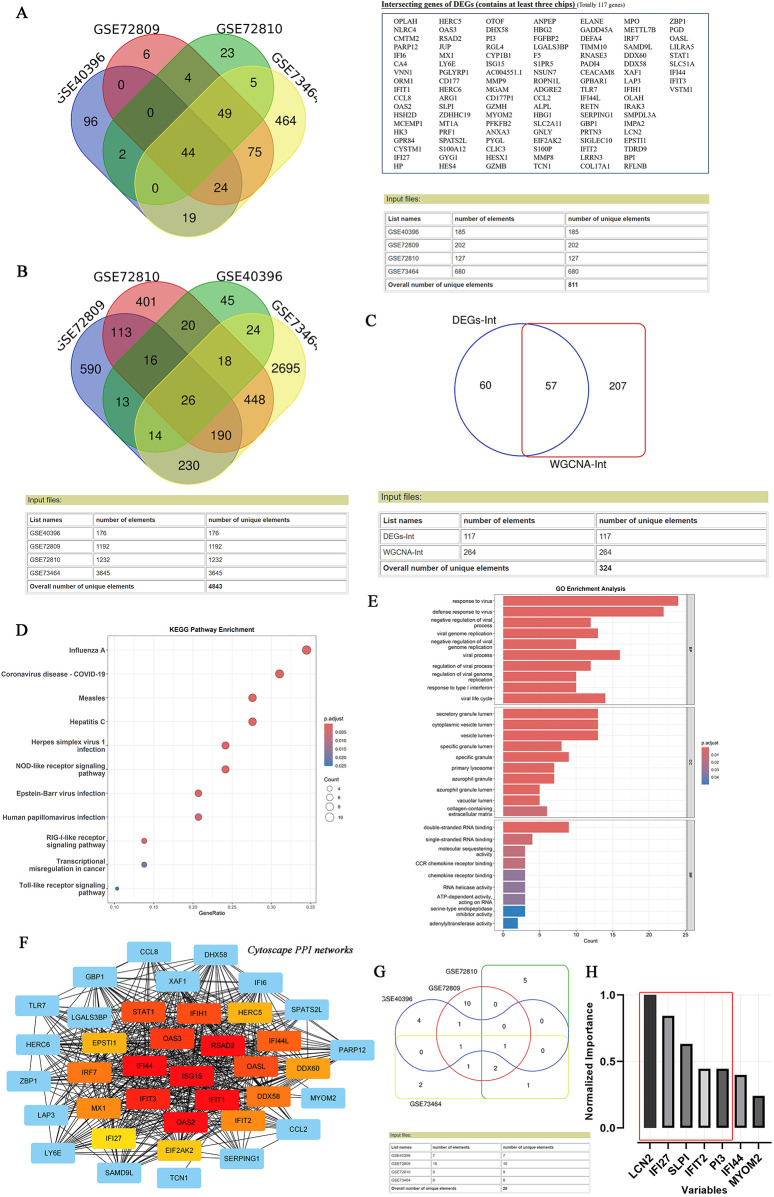
Identifying the candidate DEGs with B/V infection through DEGs analysis, WGCNA, lasso analysis and MLP. **(A)** Venn diagram of intersection differential expression genes between bacterial and viral infections. A checklist of 117 DEGs was presented, with each gene being identified in a minimum of three datasets. **(B)** Venn diagram of intersection module member genes across the multi-datasets for bacterial and viral infections, with each gene being identified in a minimum of three datasets. **(C)** An overlap of candidate genes between the DEGs and the module member genes. **(D–E)** KEGG and GO annotation (biological process, cellular component and molecular function) of 57 overlapped DEGs. **(F)** PPI network of overlapping genes analyzed using the STRING online database and visualized by CytoScape 3.9.0. **(G)** Venn diagram of intersection genes across the multi-datasets for bacterial and viral infections after Lasso analysis, with each gene being identified in a minimum of two datasets. **(H)** Variables’ significant analysis was conducted to screen the overlap of 57 candidate genes by Multilayer Perceptron (SPSS Statistics 20.0).

As depicted in the flowchart in Figure 1, the transcriptome data of 384 febrile children (definite bacterial: *n* = 135, definite viral: *n* = 249) were included in the first stage of the analysis. Volcano plots revealed 127, 202, 185 and 680 DEGs in the GSE72810, GSE72809, GSE40396 and GSE73464 datasets, respectively (Supplementary File 4). Heatmaps revealed the top 20 DEGs between children with B/V infection (Supplementary Figure File 4). To analyse the intersecting DEGs in the gene expression profiles between B/V patients, a total of 117 DEGs were displayed in a Venn diagram, with each gene being identified in a minimum of three datasets (Figure 2A). These 117 genes serve as potential host genes obtained from the DEGs analysis for subsequent research.

Subsequently, WGCNA was applied to construct scale-free co-expression networks for each dataset. The clustered modules across multiple datasets are displayed in Supplementary File 5. Module member genes in each dataset were identified via WGCNA. In total, 264 genes were represented in a Venn diagram, with each gene identified in at least three datasets (Figure 2B). An overlap of 57 candidate genes between the 117 DEGs and the 264 module member genes was subsequently identified (Figure 2C). To reduce overfitting risk in the diagnostic model, LASSO regression and variable significance analysis (MLP) were used to screen these 57 candidate genes. Concurrently, a PPI network was visualized via *Cytoscape* (Figure 2F). MLP ranked the input variables by normalized importance: LCN2 (100.0%), IFI27 (84.4%), SLPI (63.2%), IFIT2 (44.6%) and PI3 (44.5%) (Figures 2G,H). Forest plots for the candidate genes were constructed via *comprehensive meta-analysis*, providing an overview of the confidence intervals for each signature across multiple datasets (Supplementary File 4).

In addition, KEGG analysis revealed that 57 input genes are strongly associated with host immune responses to major viral infections (especially influenza A, COVID-19, and measles) and innate immunity pathways (NLR, RLR and TLR signalling). The GO results revealed that the DEGs were enriched in host responses to viruses, particularly in defence mechanisms, the regulation of viral replication and chemokine signalling, etc. (Figures 2D,E).

### 3.2 Evaluation of the five-gene host signature in the diagnosis of febrile children with B/V infection

**Figure 3:**
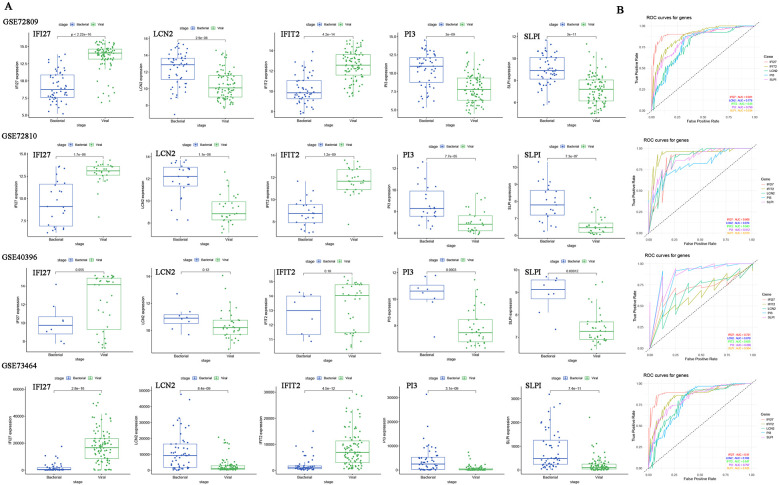
The expression levels and ROC curves of host genes signature between bacterial and viral infections across multiple datasets for diagnosis. **(A)** Expression of five diagnostic genes in B/V infections. Two-group differences were analyzed by unpaired Student's *t*-test (two-tailed). **(B)** ROC curves for each host gene in distinguishing between viral and bacterial infections. *p < 0.05, **p < 0.01, ***p < 0.001 and ns: *p* > 0.05*.*

A five-gene host signature (IFIT2, SLPI, IFI27, LCN2 and PI3) was identified through DEGs analysis, WGCNA, LASSO regression and MLP. Therefore, the combined utility of these genes for predicting bacterial/viral (B/V) infections requires further evaluation. The boxplots display the expression levels of the five diagnostic host genes in defined bacterial and viral infections (Figure 3A). The vast majority of signatures in the datasets were significantly different between the two groups (*p* *<* *0.05*), except for LCN2 and IFIT2 in GSE40396 (*p* *>* *0.05)*. To determine the diagnostic value of host gene signatures in distinguishing viral from bacterial infections, ROC curves for each gene were plotted with R (Figure 3B). These results consistently indicate that the genes IFIT2, SLPI, IFI27, LCN2, and PI3 have significant predictive value for differentiating between B/V infections in febrile children.

### 3.3 Development of ANN and RF models for diagnosing bacterial/viral (B/V) infections in febrile children

**Figure 4:**
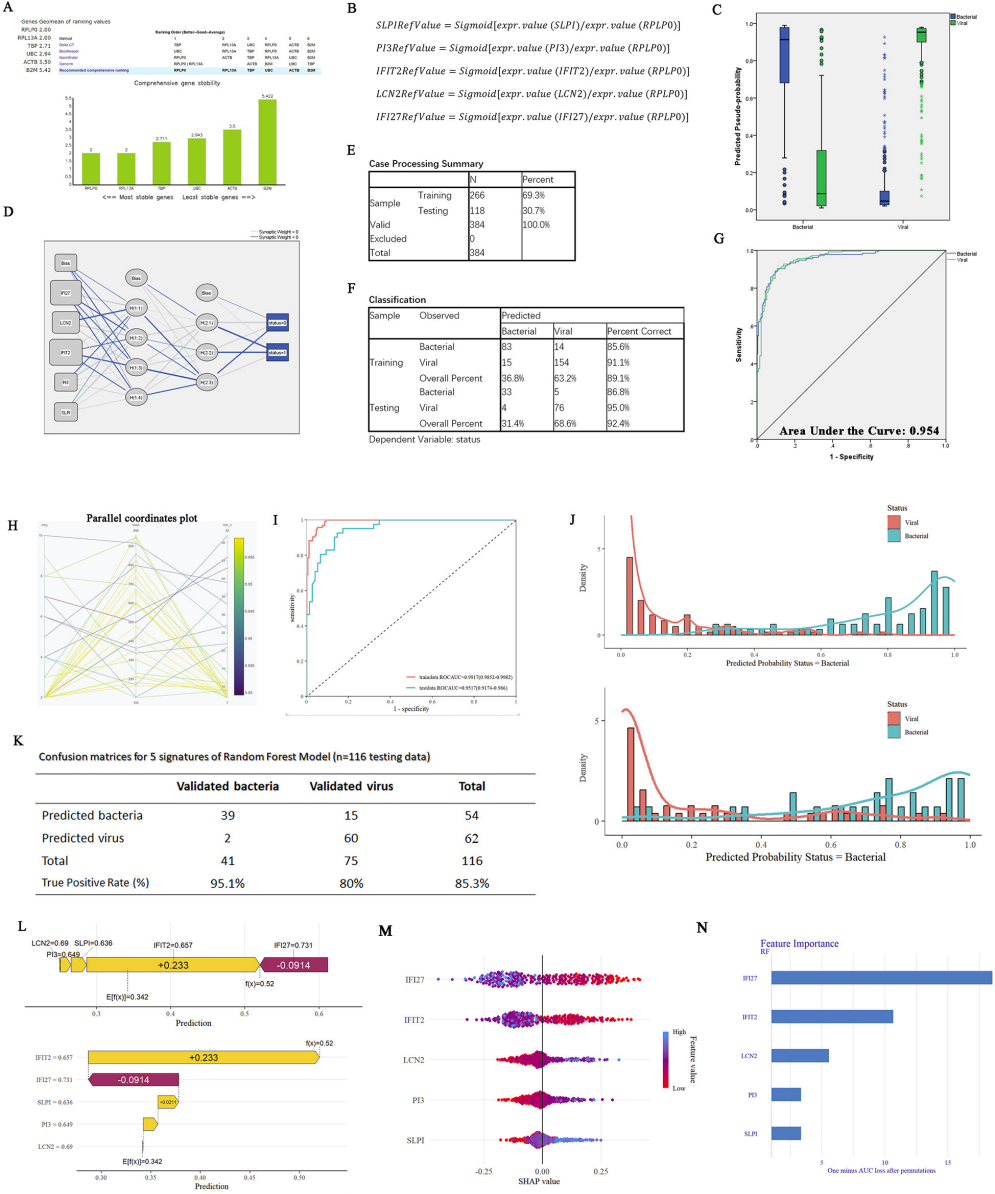
Constructions of ANN and RF models on five-gene host signature for the diagnosis of febrile children with B/V. **(A)** Genes geomean of ranking values for six candidate reference genes (B2M, TBP, ACTB, UBC, RPLP0, and RPL13A) was calculated using RefFinder. **(B)** The mathematical preprocessing formula was utilized to convert the originate data to RefValue (i). **(C)** The box plot displays the predicted pseudo-probability of B/V. **(D)** The framework of a multilayer perceptron neural network with two hidden layers: the activation function in hidden layers (Hyperbolic tangent); Activation function in output layer (Softmax); Error function in output layer (Cross-entropy). **(E)** Case processing summary for the input samples (5 variables and 1 outcomes). **(F)** Classification outcomes of the ANN model for predicting B/V infection. **(G)** ROC curves of the ANN model for five-gene host signature in differentiating between children B/V infections. **(H)** Random Forest hyperparameter optimization (mtry = 8, min_n = 14, number of trees = 694). **(I)** ROC curves of the RF model for five genes in distinguishing between B/V infections. **(J)** Compound bar charts displaying the predicted probabilities of B/V status using the ggplot2 package. **(K)** Confusion matrices for five genes on GSE40396, GSE72810, GSE72809, and GSE73464 using the RF model (*n* = 116 testing data). **(L)** A SHAP force plot for a RF model. The base value represents the average prediction of the model without considering any signature features. E[f(x)] is the probability forecast value of each host gene, yellow indicates it increases the likelihood of the predicted outcome and red indicates it decreases the likelihood of the predicted outcome. **(M**,**N)** SHAP summary plot of the 5 genes of the RF model. A higher SHAP value for the host gene leads to an increased probability of viral infection.

To mitigate the high variability inherent in sample data originating from diverse chip sources and to improve the generalization performance of the predictive model, we employed a series of specific mathematical transformations for the preprocessing of data before their incorporation into the model (Figures 4A,B). In addition, the housekeeping gene *RPLP0* was identified among the six candidate reference genes (*B2M*, *TBP*, *ACTB*, *UBC*, *RPLP0* and *RPL13A*) using *Delta CT*, *BestKeeper*, *NormFinder* and *Genorm* (Figure 4A). Following the completion of the aforementioned preparatory work, an ANN was constructed for binary classification of bacterial and viral infections using *SPSS Statistics*. This study included 384 cases, with a split of 266 for training and 118 for testing, and a multilayer perceptron neural network with two hidden layers was employed. The hidden layer adopts: the activation function in hidden layers (Hyperbolic tangent); Activation function in output layer (Softmax); Error function in output layer (Cross-entropy) (Figures 4C–G). The ANN model demonstrated high predictive performance, with correct classification rates of 92.4% accuracy, 86.8% sensitivity, and 95.0% specificity in the testing set. The ROC curve analysis further confirmed its diagnostic ability (AUC = 0.954; Figures 4F,G) (the ANN framework is provided in Supplementary File 11).

Additionally, a diagnostic prediction model for febrile children with B/V infections was constructed using the RF method (R version 4.4.1). The model consists of 694 trees in the random forest and requires at least 8 random features for each branch split (Figure 4H), with ROCAUC = 0.9917 (0.9852–0.9982) in the training data and ROCAUC = 0.9517 (0.9174–0.986) in the testing data (Figure 4I). Compound bar charts display the predicted probabilities of bacterial or viral status (Figure 4J). The accuracy rates of the RF in the confusion matrix reach 85.3% in the testing cases (*n* = 116) (Figure 4K). SHapley Additive exPlanations (SHAP) force and dependency plots of the host signatures of the five genes illustrate how each feature contributes to the final prediction of the outcome (Figures 4l–N). Specifically, IFI27 contributed the most significantly to the prediction of outcomes, followed by IFIT2 and LCN2 (Figures 4M,N).

**Figure 5:**
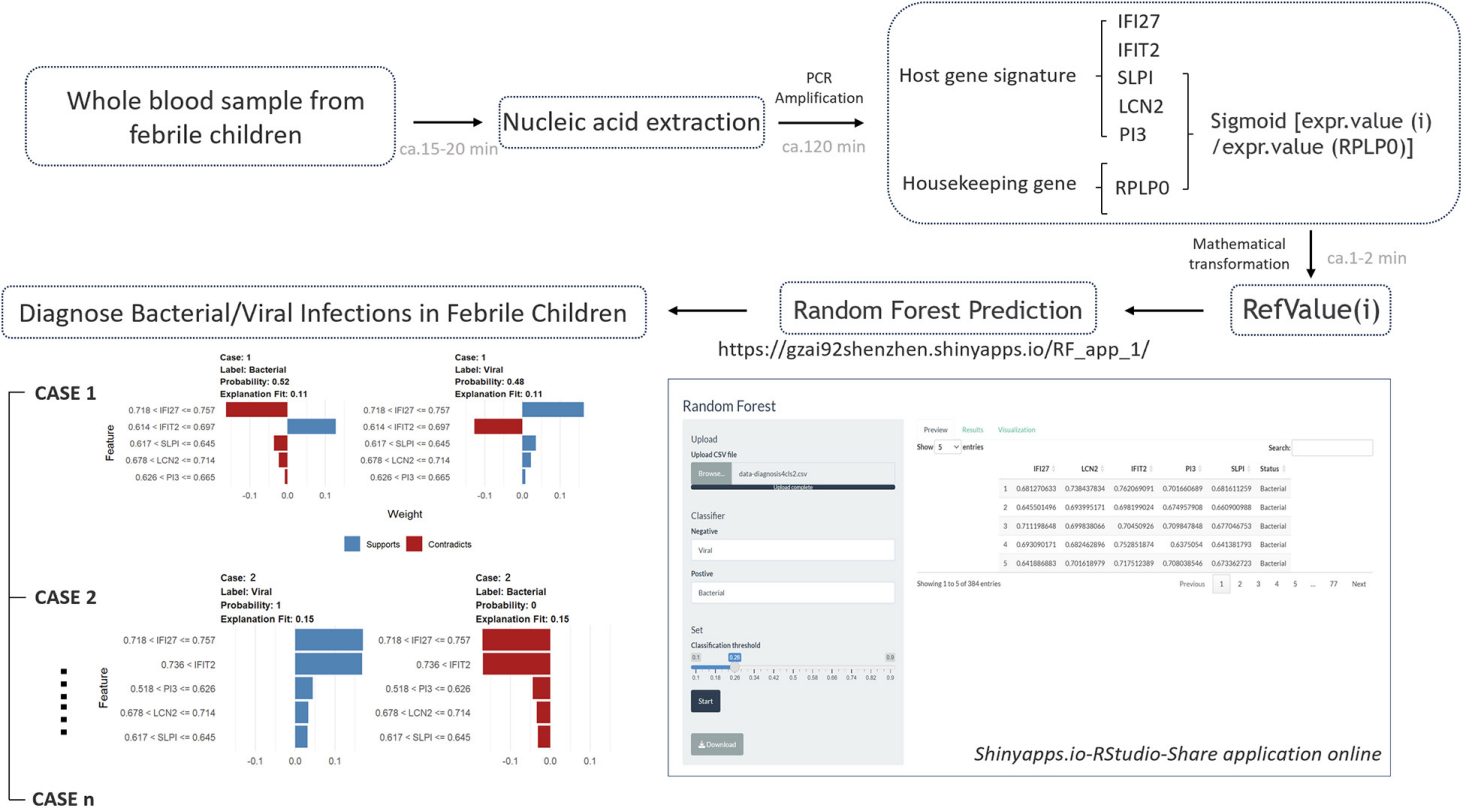
The designed testing procedure of RF prediction model for febrile children with B/V infections.

The designed testing procedure is depicted in the figure below (Figure 5). To increase the utility and practicality of the RF model for febrile children, we exported metadata (model_metadata. RData, predtrain_rf_results.rds and final_rf_model.rds) and persisted the constructed RF model object to enable reproducible deployments. These files have been transformed into RF_app.R, which enables the input of the five genes as RefValue (i) data types to make online predictions for febrile children with B/V infections: (https://gzai92shenzhen.shinyapps.io/RF_app_1/). With the increase in subsequent training data and refinement of the program, we believe that the app will achieve better predictive performance in the future.

### 3.4 Generalizing the random forest model for predicting and diagnosing different aetiological types of B/V infections

**Figure 6:**
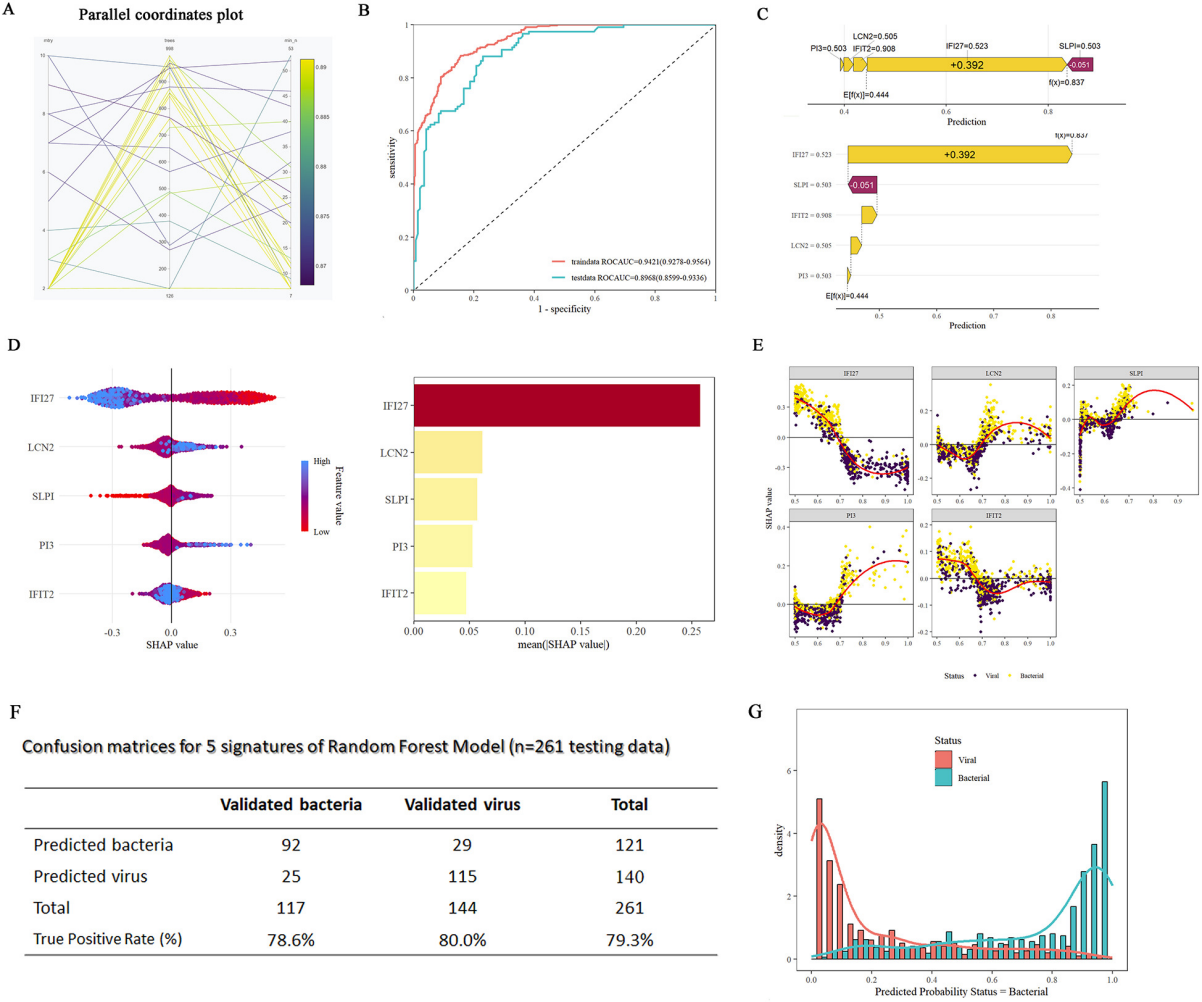
Constructions of generalized RF models on five-gene host signature for the diagnosis of patient with B/V. **(A)** RF hyperparameter optimization (mtry = 2, min_n = 40, number of trees = 729). **(B)** ROC curves of the RF model for five-gene host signature in differentiating between viral and bacterial infections. **(C)** A SHAP force plot for a RF model. The base value represents the average prediction of the model without considering any signature features. E[f(x)] is the probability forecast value of each host gene, yellow indicates it increases the likelihood of the predicted outcome and red indicates it decreases the likelihood of the predicted outcome. **(D)** SHAP summary plot of the 5 features of the RF model. A higher SHAP value for the host gene leads to an increased probability of viral infection. **(E)** SHAP dependency plots of the 5 features of the RF model. Each subfigure shows the relationship between feature and its SHAP values, along with the distribution of the SHAP value for different classification (viral vs. bacterial). **(F)** Confusion matrices for the generalized RF model using five-gene host signature on testing samples (*n* = 261, testing data). **(G)** Compound bar charts displaying the predicted probabilities of B/V status using the ggplot2 package.

Next, we generalized the RF model to enhance its predictive ability for samples with intricate background complexities in clinical practice. In addition to the previously used datasets (GSE40396, GSE72810, GSE72809, and GSE73464) involving febrile illness in children, the random forest training set included more complex datasets (*n* = 1,042: bacterial = 468 patients, viral = 574 patients) related to B/V infections. These datasets included infections caused by *Staphylococcus aureus*, *Escherichia coli* (EPEC, EAEC, and DAEC), *Salmonella*, *Shigella* unknown bacterial pneumonia, *adenovirus*, *HHV6*, *enterovirus*, *rhinovirus*, *human rotavirus*, *human norovirus*, and *influenza A pneumonia*, among others. The RF model (Figure 6A) was constructed, with ROCAUC = 0.9421 (0.9278–0.9564) for the training data and ROCAUC = 0.8968 (0.8599–0.9336) for the testing data (Figure 6B). SHAP force and dependency plots of the host signatures of the five genes illustrate how each feature contributes to the final prediction of the outcome (Figures 6C–E). Specifically, IFI27 contributed the most significantly to the prediction of outcomes, followed by LCN2 and IFIT2 (Figure 6D). The generalized RF model, as indicated in the confusion matrices, achieves 79.3% accuracy in predicting diverse aetiological types of B/V infections (Figure 6F). Compound bar charts display the predicted probabilities of bacterial or viral status (Figure 6G). These results consistently suggest that the constructed RF model has an acceptable ability to predict patients with complex aetiologies in the diagnosis of B/V infections.

## 4 Discussion

Nowadays, the early diagnosis of B/V infections among febrile children remains a formidable challenge in clinical practice. Due to the immature development of the immune system in children and the challenges in communication during medical consultation, the onset of fever in this population often proceeds rapidly and is frequently results in severe complications, such as sepsis and meningitis (30, 31). Even with the rapid development of high-throughput technologies today, most physicians still rely solely on clinical symptoms, full blood count (FBC), CRP, and PCT for diagnosing the type of infection in febrile children (11, 12). Constrained by their limited sensitivity and specificity, these diagnostic markers cannot replace the “gold standard diagnostic test” to meet clinical requirements. On the other hand, the traditional pathogen culturing method is time-consuming, while nucleic acid or serological testing requires accurate etiological hypotheses. Hence, a new diagnostic strategy based on host gene signatures has emerged: In 2021, Ravichandran et al. published a study in *EBioMedicine* introducing a 10-gene blood-based biomarker panel (Panel-VB) that demonstrated high accuracy in distinguishing disease states (32). Rao et al. presented an eight-gene signature in Cell Report Medicine in 2022 that can differentiate between intra- and extracellular bacterial infections and viral infections, with an AUROC > 0.91 (16). Xie et al. reported in BMC Pediatrics in 2023 on a five-gene signature for the early diagnosis of Kawasaki disease, with an AUROC exceeding 0.9 (33). In the same year, Habgood-Coote utilized 161 transcripts to distinguish eighteen specific diseases or causative pathogens in children, with AUROC >0.9 in the validation set (34). These studies collectively highlight the potential application of the host gene signatures in the early diagnosis of diseases. In our study, by collecting multiple datasets from febrile children, we identified intersecting genes between DEGs and WGCNA. Through PPI analysis, immune infiltration analysis, Lasso regression, mathematical transformations, and housekeeping gene calibration, high-accuracy models for ANN and RF were successfully constructed, achieving the AUROC of 0.954, 0.9917_(RF training)_ and 0.9517_(R*F* testing)_, respectively. Our models achieve acceptable accuracy and are suitable for predicting the infection type in febrile children.

The number of host gene signatures used to construct predictive models is not necessarily fewer for better performance, as human physiological regulation involves complex mechanisms. Non-specific genes identified from host immune may lack the specificity required for accurate prediction of infection types. Conversely, too many features involved in the model will cause overfitting, which may lead to higher detection costs and reduce the model's generalization ability. Therefore, one of the innovations in this study is the use of a concatenated approach that combines WGCNA, Lasso, and MLP variable significance analysis to refine the predictive features. Eventually five-gene host signature were identified for the diagnosis of febrile children with B/V infections. Our five genes included three genes overexpressed in bacterial infections (LCN2, PI3, and SLPI) and two overexpressed in viral infections (IFI27 and IFIT2). More information on these five genes were provided in Supplementary File 9. LCN2 and SLPI have been previously published in *Clinical Infectious Diseases* for distinguishing bacterial from viral pediatric clinical pneumonia in a malaria-endemic setting (achieved >90% sensitivity and >80% specificity) (35). IFI27 has been mentioned as a potential diagnostic marker for respiratory syncytial virus infection in preterm infants; however, its predictive efficacy was not evaluated in the study (36, 37). Other host genes involved in children B/V prediction include: ADM, ALPL, HK3, MMP9, S100A12, HP, LTF, MPO, MMP8, PGLYRP1, RETN, SERPINA1, S100A9, IFI44l, FAM89A, etc. In this study, we innovatively trained artificial neural network (ANN) and random forest (RF) models using a novel five-gene host signature to diagnose bacterial/viral (B/V) infections in children. The RF model achieved an AUC of 0.9917 in training and 0.9517 in testing, while the ANN model achieved an AUC of 0.954 in testing.

Previous studies have mentioned a problem that the high cost and the need for dedicated skills greatly hinder the development of host-RNA signature diagnostics in clinical application (30). In our study, the RefValue (i), serving as the core feature values of the RF model, is derived through lasso regression, housekeeping gene calibration, and mathematical transformations. By utilizing the RefValue (i), the operation of the RF model requires the input of only five relative quantification ratios, which can be completed using PCR within 2 h. This design confers an advantage in handling complex background samples and enhancing the model generalizability and clinical application.

As known, batch effect removal is beneficial for multi-dataset analysis because it enhances inter-groups comparability and eliminates technical variations. However, these methods (such as COMBAT and COCONUT) may remove some biological variations between the different groups and do not always provide better results for the prediction (16). One of the interesting aspects of our study design is that we did not involve conventional batch effect removal at the beginning of the DEGs and WGCNA analyses. As shown in the flowchart, the data from GSE40396, GSE72809, GSE72810 and GSE7346 are analysed independently for differential expression, and an intersection analysis is subsequently conducted to integrate these individual differences into a set of candidate features. Such approach is advantage because more potentially predictive features have been retained throughout the analysis. On another hand, it is worth saying that an immune infiltration analysis has been conducted to examine the differences in 22 types of immune cells between febrile children with bacterial and viral infections. In this study, macrophages M0, resting NK cells, neutrophils and naive CD4T cells were consistently identified as positive in the forest plots (Supplementary Files 7, 8). Among these, macrophages M0 and NK cells resting are mentioned for the first time as predictors of the infection type in febrile children, that providing a new direction for exploring the underlying mechanisms.

Based on a five-gene RF model, we developed a new diagnostic classifier that can correctly determine the infection type of febrile children in 384 cases, achieving 85.3% in testing cases (*n* = 116) with AUC_testing_ = 0.9517. In terms of children B/V infection, there were relatively obvious improvements when compared to the FBC, CRP and procalcitonin methods, as well as similar published models [*Herberg* DRS: AUC of 0.825 (0.691–0.959); *Channon*'s multiclass diagnosis model: AUC of 0.825–0.867; *Jackson*'s multi-platform approach: AUROC between 89·4% and 93·6%] (6, 38–40). In this study, we present the diagnostic RF models based on five-gene host signature with RefValue (LCN2, PI3, SLPI, IFI27 and IFIT2) in whole blood samples that could correctly distinguish B/V infections in febrile children (85.3% accuracy, 95.1% sensitivity, 80.0% specificity), meeting/exceeding the Foundation for Innovative New Diagnostics (FIND)-sponsored expert guidelines for diagnostic sensitivity/specificity to differentiate between bacterial and non-bacterial infections (35). In addition, we provided a RF model for justifying a given sample with complex etiologies in more than 1,084 cases in all. Applicable to diagnosis bacterial infection including: *Staphylococcus aureus*, *Escherichia coli* (EPEC, EAEC, DAEC), *Salmonella*, *Shigella* unknown bacterial pneumonia; and applicable to diagnosis viral infection including: *Adenovirus*, *HHV6*, *Enterovirus*, *Rhinoviru*s, *human Rotavirus*, *human Norovirus*, *influenza A pneumonia*, among others, achieving ROCAUC_training_ = 0.9421 (0.9278–0.9564) and ROCAUC_testing_ = 0.8968 (0.8599–0.9336). Our study assesses the clinical effectiveness of the RF model for guiding decision-making regarding the infection type in febrile children, in terms of whether or not to prescribe antibiotics or antiviral treatments.

Limitations of the study: (1) After the five-gene host signature RF models were finished being trained, we did not collect whole blood samples from febrile children in representative districts to determine their clinical practicality. However, in the testing phase, we utilized 30% of randomly generated cases for validation, which achieved a ROCAUC of 0.9517, indicating the acceptable diagnosis value for children B/V infection of the model. (2) Limited by the insufficient number of representative samples, co-infections cases were not involved in the RF model in the study. Actually, we identified that LCN2, PI3, and SLPI were up-regulated in bacterial infections, and IFI27 and IFIT2 were up-regulated in viral infections in the study, which may be helpful in assisting with the diagnosis of B/V co-infection in children. In the future, more complex etiologies and noise handling will be considered for the improvement of the RF model.

## 5 Conclusions

In this study, a five-gene host signature (IFIT2, SLPI, IFI27, LCN2, and PI3) was identified through DEGs analysis, WGCNA, LASSO regression, and variable significance analysis. An RF model constructed using this signature achieved 85.3% accuracy, 95.1% sensitivity, and 80.0% specificity in diagnosing B/V infections in febrile children. The constructed ANN model achieved 92.4% accuracy, 86.8% sensitivity, and 95.0% specificity. These results provide guidance for antibiotic/antiviral treatment decisions in children with unknown infection types.

## Data Availability

Publicly available datasets were analyzed in this study. This data can be found here: datasets involved in the study including: GSE72809, GSE72810, GSE40396, GSE73464, GSE40012, GSE69529, GSE63990, GSE42026 and GSE60244. These data are available in the NCBI-GEO database (https://www.ncbi.nlm.nih. gov/geo/).

## References

[1] Hao R Saleh M Liang T Molyneaux N Gordon I Anyachebelu C The prevalence of serious bacterial infections in neutropenic immunocompetent febrile children. Am J Emerg Med. (2021) 45:1–6. 10.1016/j.ajem.2021.02.017 33639293

[2] Rose E. Pediatric fever. Emerg Med Clin North Am. (2021) 39(3):627–39. 10.1016/j.emc.2021.04.011 34215406

[3] Graaf S Keuning MW Pajkrt D Plötz FB. Fever without a source in children: international comparison of guidelines. World J Pediatr. (2023) 19(2):120–8. 10.1007/s12519-022-00611-8 36287322 PMC9928815

[4] Schlapbach LJ Watson RS Sorce LR Argent AC Menon K Hall MW International consensus criteria for pediatric sepsis and septic shock. JAMA. (2024) 331(8):665–74. 10.1001/jama.2024.0179 38245889 PMC10900966

[5] Kang Y Chen S Chen Y Tian L Wu Q Zheng M Alterations of fecal antibiotic resistome in COVID-19 patients after empirical antibiotic exposure. Int J Hyg Environ Health. (2022) 240:113882. 10.1016/j.ijheh.2021.113882 34915282 PMC8664710

[6] Murray CJL. Global burden of bacterial antimicrobial resistance 1990–2021: a systematic analysis with forecasts to 2050. Lancet. (2024) 404(10459):1199–226. 10.1016/s0140-6736(24)01867-1 39299261 PMC11718157

[7] Hagedoorn NN Borensztajn DM Nijman R Balode A von Both U Carrol ED Variation in antibiotic prescription rates in febrile children presenting to emergency departments across Europe (MOFICHE): a multicentre observational study. PLoS Med. (2020) 17(8):e1003208. 10.1371/journal.pmed.1003208 32813708 PMC7444592

[8] Hasegawa T Aomatsu K Nakamura M Aomatsu N Aomatsu K. Cytomegalovirus colitis followed by ischemic colitis in a non-immunocompromised adult: a case report. World J Gastroenterol. (2015) 21(12):3750–4. 10.3748/wjg.v21.i12.3750 25834346 PMC4375603

[9] Nguyen AV Orlofsky A Pubill K Tawde M Li G Mata D Loop-mediated isothermal amplification (LAMP) as a rapid, affordable and effective tool to involve students in undergraduate research. Front Microbiol. (2020) 11:603381. 10.3389/fmicb.2020.603381 33362748 PMC7756096

[10] Santotoribio JD Nuñez-Jurado D Lepe-Balsalobre E. Evaluation of routine blood tests for diagnosis of suspected coronavirus disease 2019. Clin Lab. (2020) 66(9):1867–75. 10.7754/Clin.Lab.2020.200522 32902237

[11] Lien F Lin HS Wu YT Chiueh TS. Bacteremia detection from complete blood count and differential leukocyte count with machine learning: complementary and competitive with C-reactive protein and procalcitonin tests. BMC Infect Dis. (2022) 22(1):287. 10.1186/s12879-022-07223-7 35351003 PMC8962279

[12] Van Duffel L Yansouni CP Jacobs J Van Esbroeck M Ramadan K Buyze J Accuracy of C-reactive protein and procalcitonin for diagnosing bacterial infections among subjects with persistent fever in the tropics. Open Forum Infect Dis. (2022) 9(9):ofac434. 10.1093/ofid/ofac434 36092831 PMC9454028

[13] Papan C Argentiero A Porwoll M Hakim U Farinelli E Testa I A host signature based on TRAIL, IP-10, and CRP for reducing antibiotic overuse in children by differentiating bacterial from viral infections: a prospective, multicentre cohort study. Clin Microbiol Infect. (2022) 28(5):723–30. 10.1016/j.cmi.2021.10.019 34768022

[14] Herberg JA Kaforou M Gormley S Sumner ER Patel S Jones KD Transcriptomic profiling in childhood H1N1/09 influenza reveals reduced expression of protein synthesis genes. J Infect Dis. (2013) 208(10):1664–8. 10.1093/infdis/jit348 23901082 PMC3805235

[15] Herberg JA Kaforou M Wright VJ Shailes H Eleftherohorinou H Hoggart CJ Diagnostic test accuracy of a 2-transcript host RNA signature for discriminating bacterial vs. viral infection in febrile Children. JAMA. (2016) 316(8):835–45. 10.1001/jama.2016.11236 27552617 PMC5997174

[16] Rao AM Popper SJ Gupta S Davong V Vaidya K Chanthongthip A A robust host-response-based signature distinguishes bacterial and viral infections across diverse global populations. Cell Rep Med. (2022) 3(12):100842. 10.1016/j.xcrm.2022.100842 36543117 PMC9797950

[17] Ko ER Reller ME Tillekeratne LG Bodinayake CK Miller C Burke TW Host-response transcriptional biomarkers accurately discriminate bacterial and viral infections of global relevance. Sci Rep. (2023) 13(1):22554. 10.1038/s41598-023-49734-6 38110534 PMC10728077

[18] Al-Barakati H Newman RH Kc DB Poole LB. Bioinformatic analyses of peroxiredoxins and RF-PRX: a random forest-based predictor and classifier for PRXS. Methods Mol Biol. (2022) 2499:155–76. 10.1007/978-1-0716-2317-6_8 35696080 PMC9844236

[19] Jose B Gopinath S Vijayanatha Kurup A Nair M Pillai A Kumar A Improving the accuracy of epileptogenic zone localization in stereo EEG with machine learning algorithms. Brain Res. (2023) 1820:148546. 10.1016/j.brainres.2023.148546 37633355

[20] Colvin JM Muenzer JT Jaffe DM Smason A Deych E Shannon WD Detection of viruses in young children with fever without an apparent source. Pediatrics. (2012) 130(6):e1455–62. 10.1542/peds.2012-1391 23129086 PMC3507256

[21] Hu X Yu J Crosby SD Storch GA. Gene expression profiles in febrile children with defined viral and bacterial infection. Proc Natl Acad Sci U S A. (2013) 110(31):12792–7. 10.1073/pnas.1302968110 23858444 PMC3732941

[22] Wright VJ Herberg JA Kaforou M Shimizu C Eleftherohorinou H Shailes H Diagnosis of Kawasaki disease using a minimal whole-blood gene expression signature. JAMA Pediatr. (2018) 172(10):e182293. 10.1001/jamapediatrics.2018.2293 30083721 PMC6233768

[23] Parnell GP McLean AS Booth DR Armstrong NJ Nalos M Huang SJ A distinct influenza infection signature in the blood transcriptome of patients with severe community-acquired pneumonia. Crit Care. (2012) 16(4):R157. 10.1186/cc11477 22898401 PMC3580747

[24] DeBerg HA Zaidi MB Altman MC Khaenam P Gersuk VH Campos FD Shared and organism-specific host responses to childhood diarrheal diseases revealed by whole blood transcript profiling. PLoS One. (2018) 13(1):e0192082. 10.1371/journal.pone.0192082 29377961 PMC5788382

[25] Tsalik EL Henao R Nichols M Burke T Ko ER McClain MT Host gene expression classifiers diagnose acute respiratory illness etiology. Sci Transl Med. (2016) 8(322):322ra311. 10.1126/scitranslmed.aad6873 PMC4905578 26791949

[26] Suarez NM Bunsow E Falsey AR Walsh EE Mejias A Ramilo O. Superiority of transcriptional profiling over procalcitonin for distinguishing bacterial from viral lower respiratory tract infections in hospitalized adults. J Infect Dis. (2015) 212(2):213–22. 10.1093/infdis/jiv047 25637350 PMC4565998

[27] Newman AM Steen CB Liu CL Gentles AJ Chaudhuri AA Scherer F Determining cell type abundance and expression from bulk tissues with digital cytometry. Nat Biotechnol. (2019) 37(7):773–82. 10.1038/s41587-019-0114-2 31061481 PMC6610714

[28] Steen CB Liu CL Alizadeh AA Newman AM. Profiling cell type abundance and expression in bulk tissues with CIBERSORTx. Methods Mol Biol. (2020) 2117:135–57. 10.1007/978-1-0716-0301-7_7 31960376 PMC7695353

[29] Xie F Wang J Zhang B. Reffinder: a web-based tool for comprehensively analyzing and identifying reference genes. Funct Integr Genomics. (2023) 23(2):125. 10.1007/s10142-023-01055-7 37060478

[30] Buonsenso D Sodero G Valentini P. Transcript host-RNA signatures to discriminate bacterial and viral infections in febrile children. Pediatr Res. (2022) 91(2):454–63. 10.1038/s41390-021-01890-z 34912024

[31] Sutiman N Yao SHW Goh SSM Sultana R Chong SL. Protocol for the diagnostic performance of C reactive protein, procalcitonin and interleukin-6 for serious bacterial infections among children ≤36 months old presenting with fever without source: a systematic review and meta-analysis. BMJ Paediatr Open. (2024) 8(1):1–5. 10.1136/bmjpo-2023-002237 PMC10952928 38499348

[32] Ravichandran S Banerjee U Dr GD Kandukuru R Thakur C Chakravortty D VB(10), a new blood biomarker for differential diagnosis and recovery monitoring of acute viral and bacterial infections. EBioMedicine. (2021) 67:103352. 10.1016/j.ebiom.2021.103352 33906069 PMC8099739

[33] Xie Y Shi H Han B. Bioinformatic analysis of underlying mechanisms of Kawasaki disease via weighted gene correlation network analysis (WGCNA) and the least absolute shrinkage and selection operator method (LASSO) regression model. BMC Pediatr. (2023) 23(1):90. 10.1186/s12887-023-03896-4 36829193 PMC9951419

[34] Habgood-Coote D Wilson C Shimizu C Barendregt AM Philipsen R Galassini R Diagnosis of childhood febrile illness using a multi-class blood RNA molecular signature. Med. (2023) 4(9):635–54.5. 10.1016/j.medj.2023.06.007 37597512

[35] Gillette MA Mani DR Uschnig C Pellé KG Madrid L Acácio S Biomarkers to distinguish bacterial from viral pediatric clinical pneumonia in a malaria-endemic setting. Clin Infect Dis. (2021) 73(11):e3939–48. 10.1093/cid/ciaa1843 33534888 PMC8653634

[36] Gao J Zhu X Wu M Jiang L Wang F He S. IFI27 may predict and evaluate the severity of respiratory syncytial virus infection in preterm infants. Hereditas. (2021) 158(1):3. 10.1186/s41065-020-00167-5 33388093 PMC7778825

[37] Yasrebi H. Comparative study of joint analysis of microarray gene expression data in survival prediction and risk assessment of breast cancer patients. Brief Bioinform. (2016) 17(5):771–85. 10.1093/bib/bbv092 26504096 PMC5863785

[38] Channon-Wells S Habgood-Coote D Vito O Galassini R Wright VJ Brent AJ Integration and validation of host transcript signatures, including a novel 3-transcript tuberculosis signature, to enable one-step multiclass diagnosis of childhood febrile disease. J Transl Med. (2024) 22(1):802. 10.1186/s12967-024-05241-4 39210372 PMC11360490

[39] Dittrich S Tadesse BT Moussy F Chua A Zorzet A Tängdén T Target product profile for a diagnostic assay to differentiate between bacterial and non-bacterial infections and reduce antimicrobial overuse in resource-limited settings: an expert consensus. PLoS One. (2016) 11(8):e0161721. 10.1371/journal.pone.0161721 27559728 PMC4999186

[40] Jackson HR Zandstra J Menikou S Hamilton MS McArdle AJ Fischer R A multi-platform approach to identify a blood-based host protein signature for distinguishing between bacterial and viral infections in febrile children (PERFORM): a multi-cohort machine learning study. Lancet Digit Health. (2023) 5(11):e774–85. 10.1016/s2589-7500(23)00149-8 37890901

