# Supplementary material for: Development of a host-signature-based machine learning model to diagnose bacterial and viral infections in febrile children
Source: Front Pediatr. 2025 Aug 6;13:1608812. doi: 10.3389/fped.2025.1608812 (PMC12364931; doi:10.3389/fped.2025.1608812)
Supplement: Supplementary file 1 [file Datasheet1.docx]

**Supplementary information**

**Development of a Host-Signature-Based Machine Learning Model to Diagnose Bacterial and Viral Infections in Febrile Children**

Fang Bai^1#^, Zelong Gong^2#^, Dong Cui^2#^, Xiaomei Zhang^2^, Wenteng Hong^2^, Yi Gao^2^, Kai Lin^2^, Weijie Chen^1^, Lu Li^1^, Juan Huang^1^, Biying Zheng^1^, Junfa Xu^1^*, Na Xiao^2^*

**Supplementary file 1 Table:** **Datasets and definite diagnosis included in the study.**

|  | GSE72809 | GSE72810 | GSE40396 | GSE73464 | Total |
| --- | --- | --- | --- | --- | --- |
| Bacterial infection | 52 | 23 | 8 | 52 | 135 |
| *Staphylococcus aureus* |  |  | 4 |  |  |
| *Escherichia coli* |  |  | 2 |  |  |
| *Salmonella* |  |  | 1 |  |  |
|  |  |  |  |  |  |
| Viral infection | 92 | 28 | 35 | 94 | 249 |
| *Adenovirus* |  |  | 11 |  |  |
| HHV6 |  |  | 10 |  |  |
| *Enterovirus* |  |  | 6 |  |  |
| *Rhinovirus* |  |  | 8 |  |  |
|  |  |  |  |  |  |

**Supplementary file 2:** Evaluation of sample distribution in multi datasets.


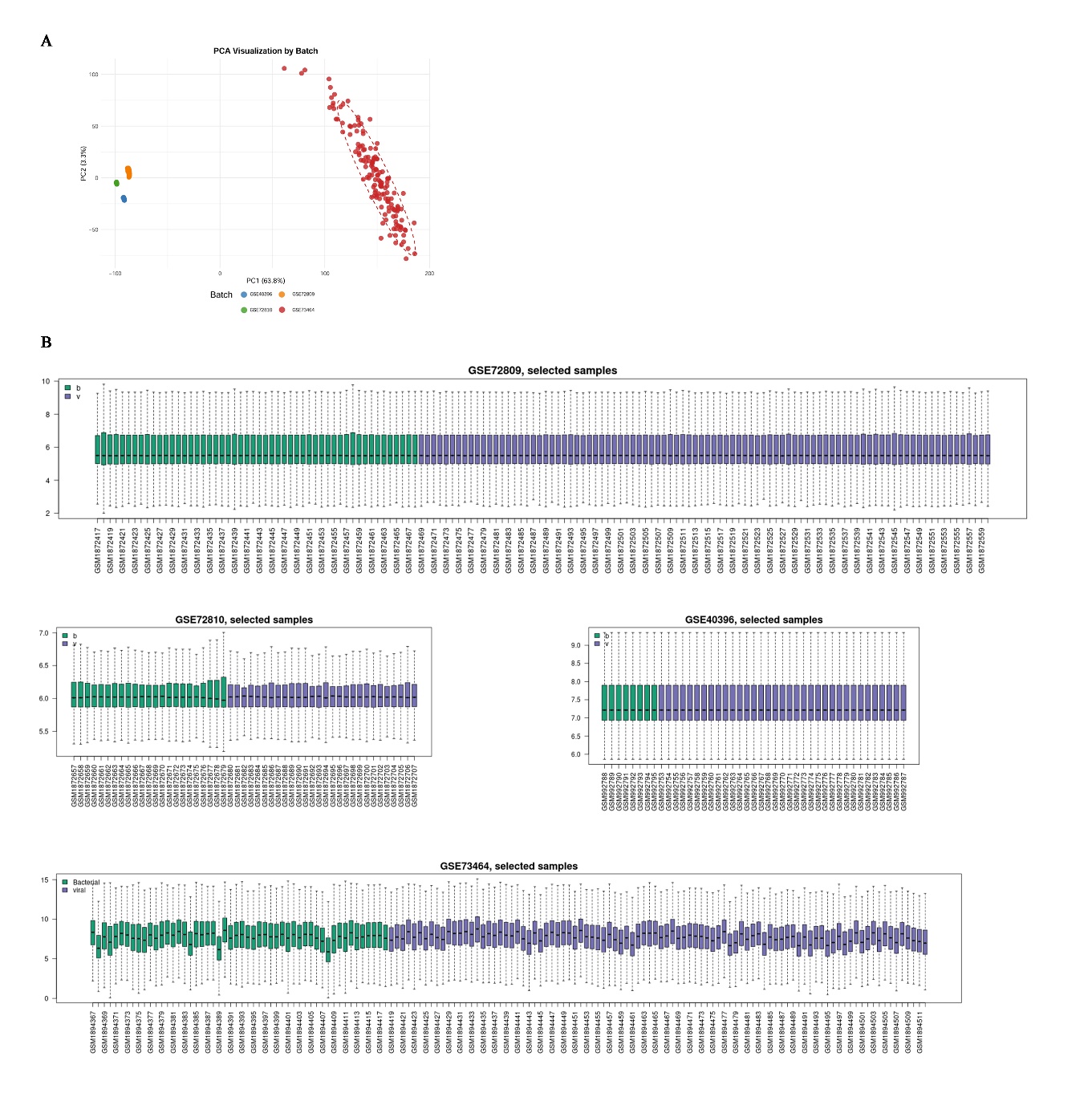


**Supplementary Fig. 2** Evaluation of sample distribution in multi datasets. (A) Principal component analysis of samples in GSE72809, GSE72810, GSE40396 and GSE73464 datasets. (B) box plots of normalized or unnormalized samples in GSE72809, GSE72810, GSE40396 and GSE73464 datasets.

**Supplementary file 3 Methods**

**3.1 Intersecting DEGs analysis of multi-dataset**

For analyzing and visualizing the transcriptomic data from public database, necessary R (version 4.4.1), *Bioconductor* packages *limma*, *DESeq2* and *ggplot* were applied in the environment. Converting the transcriptomic data matrix into the input matrix for downstream analyses. Log transformation, model fitting, and selection of significantly DEGs were involved in *limma* packages. *ggplot2* package and the functions *(plot*, *points*, *abline*, *title*) were applied to generate the volcano plot. TBtools-II v2.119 software was applied to generate heatmap. *Bioinformatics & Evolutionary Genomics* online software (http://bioinformatics.psb.ugent.be/webtools/Venn/) was used for Venn diagram analysis. Intersecting DEGs are those that exhibit statistically significant differences when identified in more than three microarrays.

**3.2 Weighted gene co-expression network analysis**

Describing the patterns of co-expression genes between bacterial and viral infection, WGCNA are generally stable and could effectively capture subtle changes in gene expression patterns. Following by data preparation, sample clustering, soft threshold selection, co-expression network construction and module membership analysis, the positively/negatively related modules and the related genes could be obtained for the subsequent analysis. R packages (*biocManager*, *WGCNA*) were involved during analysis. *hmisc*, *fastcluster* and *dynamicTreeCut* packages were utilized for hierarchical clustering and modules identification. *ggplot2* package was applied to generate plots.

**3.3 Obtaining the candidate genes**

*Bioinformatics & Evolutionary Genomics* was employed to generate the intersecting genes of DEGs and WGCNA output results. For avoiding the risk of overfitting, least absolute shrinkage and selection operator (LASSO) was conducted to reduce the variables by penalizing the regression coefficients with L1 penalty. *glmnet* package was used for variable selection in high-dimensional datasets. SPSS 20.0 was applied to independent variable important analysis. Forest plots were generated utilizing *Comprehensive Meta Analysis* V3 to integrate the specific gene (IFI27, IFIT2, LCN2, SLPI and PI3) expression patterns across multiple datasets. Protein-protein interaction network was constructed through the STRING database online tool (http://string-db.org, version 12.0). Node intersecting genes were screened by MCC using *cytoHubba* and visualized by *CytoScape* 3.9.0.

**3.4 Immune infiltration analysis**

CIBERSORTx developed by *Alizadeh* Lab and *Newman* Lab, provides 22 human immune cell types proportions (B cells naïve, B cells memory, Plasma cells, T cells CD8, T cells CD4 naïve, T cells CD4 memory resting, T cells CD4 memory activated, T cells follicular helper, Tregs, T cells gamma delta, NK cells resting, NK cells activated, Monocytes, Macrophages M0, Macrophages M1, Macrophages M2, Dendritic cells resting, Dendritic cells, activated Mast cells resting, Mast cells activated, Eosinophils, Neutrophils) through the input of gene expression profiling (https://cibersortx.stanford.edu/)(Newman et al., 2019; Steen et al., 2020). Analysis Module: Input Cell Fractions, Signature matrix file: LM22 (22 immune cell type), Disable quantile normalization, Permutations for significance analysis: 100 permutations. *corrplot, limma*, *vioplot* and *ggplot2* packages were applied to generate bar plots and violin plots for visualization. *Comprehensive Meta Analysis V3* was utilized to integrate the cell types with potentially predictive value across multiple datasets. *rms* package was used to construct nomogram, which could predict the probability of B/V infection through the corresponding values on each predictive cell type.

**Supplementary file 4 Volcano plot of the DEGs identified in multi-datasets
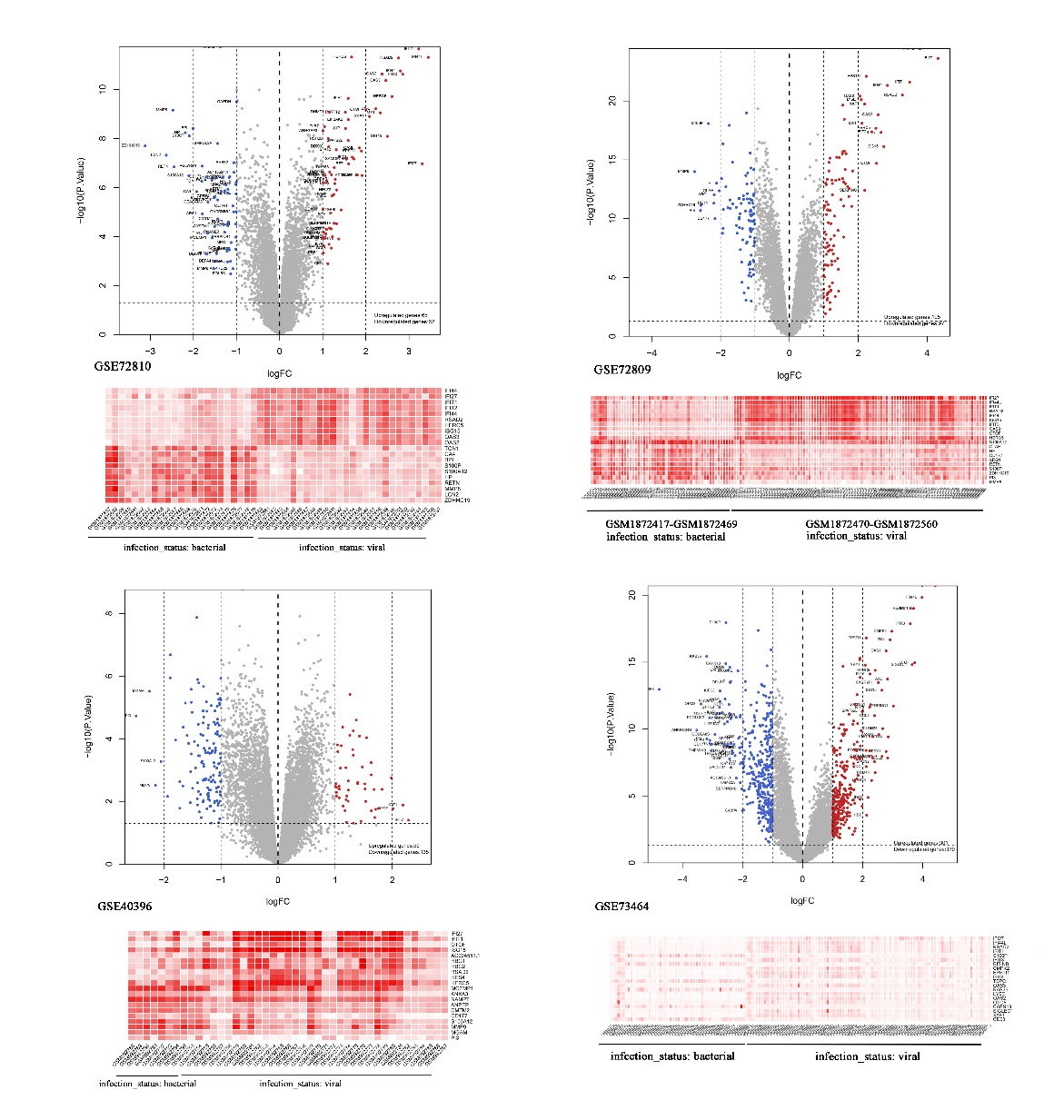
**

**Supplementary Fig. 4** Identifying the intersecting DEGs with B/V infection. Volcano plot illustrating the DEGs identified in GSE72810, GSE72809, GSE40396, and GSE73464 datasets, *limma* and *ggplot* packages were applied for volcano plot construction. DEGs criteria: abs_LogFC >1*, p<0.05* in GSE72810, GSE72809, GSE73464and GSE40396. (The red point in the plot represents the upregulated DEGs. The blue point represents the downregulated DEGs, and the grey point in the plot represents no significant genes).

**
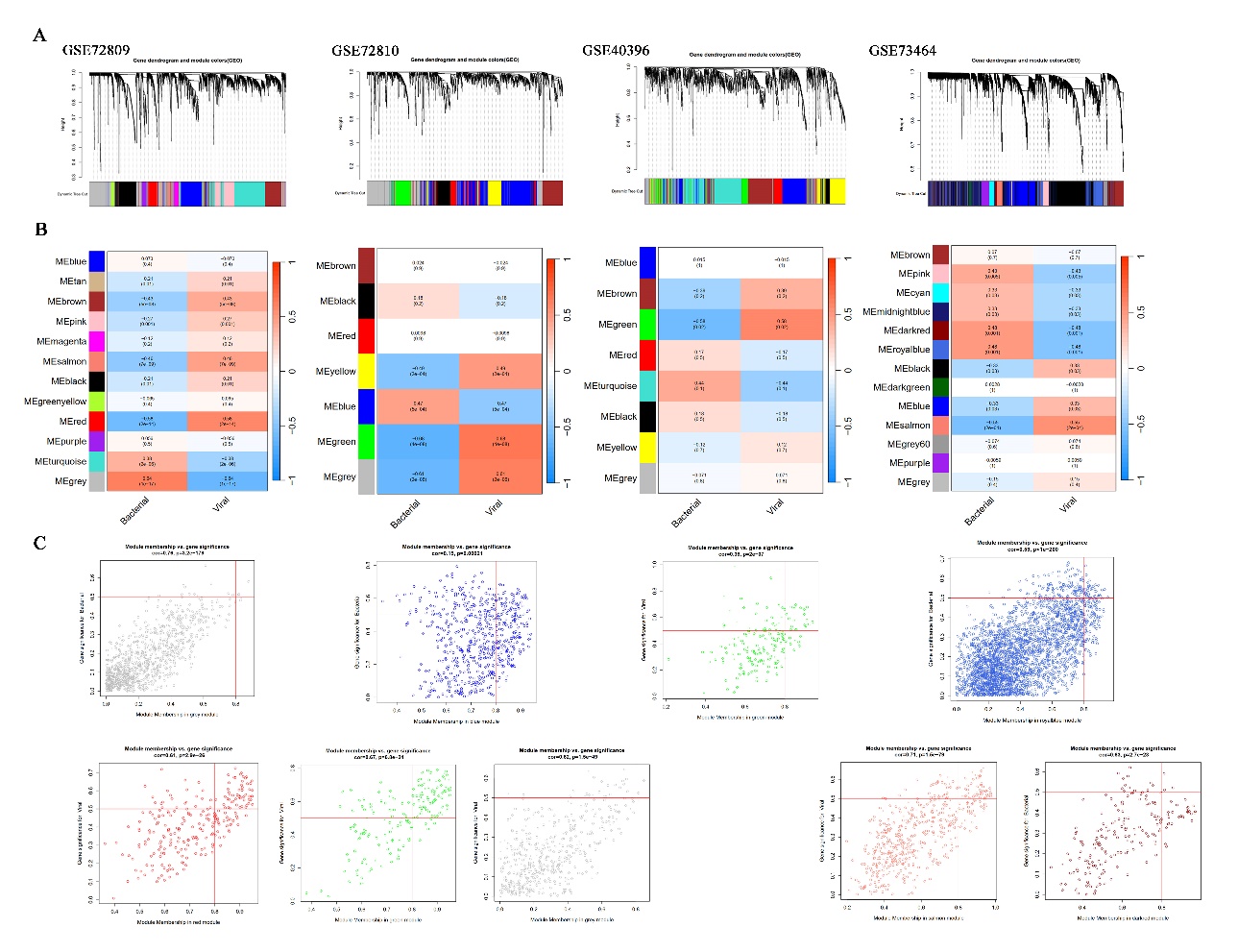
****Supplementary file 5 The WGCNA analysis of multi-datasets**

**Supplementary Fig. 5** The WGCNA analysis of multi-datasets and identification of overlapped candidate genes. (A) The cluster dendrogram of WGCNA in GSE72809 (GSM1872417-GSM1872468, GSM1872469-GSM1872560), GSE72810 (GSM1872657-GSM1872679, GSM1872680-GSM1872707), GSE40396 (GSM992788-GSM992795, GSM992753-GSM992787), GSE73464 (GSM1894367-GSM1894418, GSM1894419-GSM1894470) datasets. (B) The clustered modules of WGCNA in multi-datasets. (C) Correlation between module member genes (MEred, MEgrey, MEgreen, MEblue, MEsalmon, MEdarked, MEroyalblue, etc.) and B/V infection traits.

**Supplementary file 6 The Lasso analysis for signature genes**

**Supplementary Fig. 6** The Lasso and MLP machine algorithms for signature genes. (A) LASSO plots for the models in GSE40396, GSE72809, GSE72810, and GSE7346, respectively, with error bars denoting standard errors. (B) Forest plots of IFIT2, SLPI, IFI27, LCN2 and PI3, providing an overview of the confidence intervals for each candidate signature across multiple datasets.
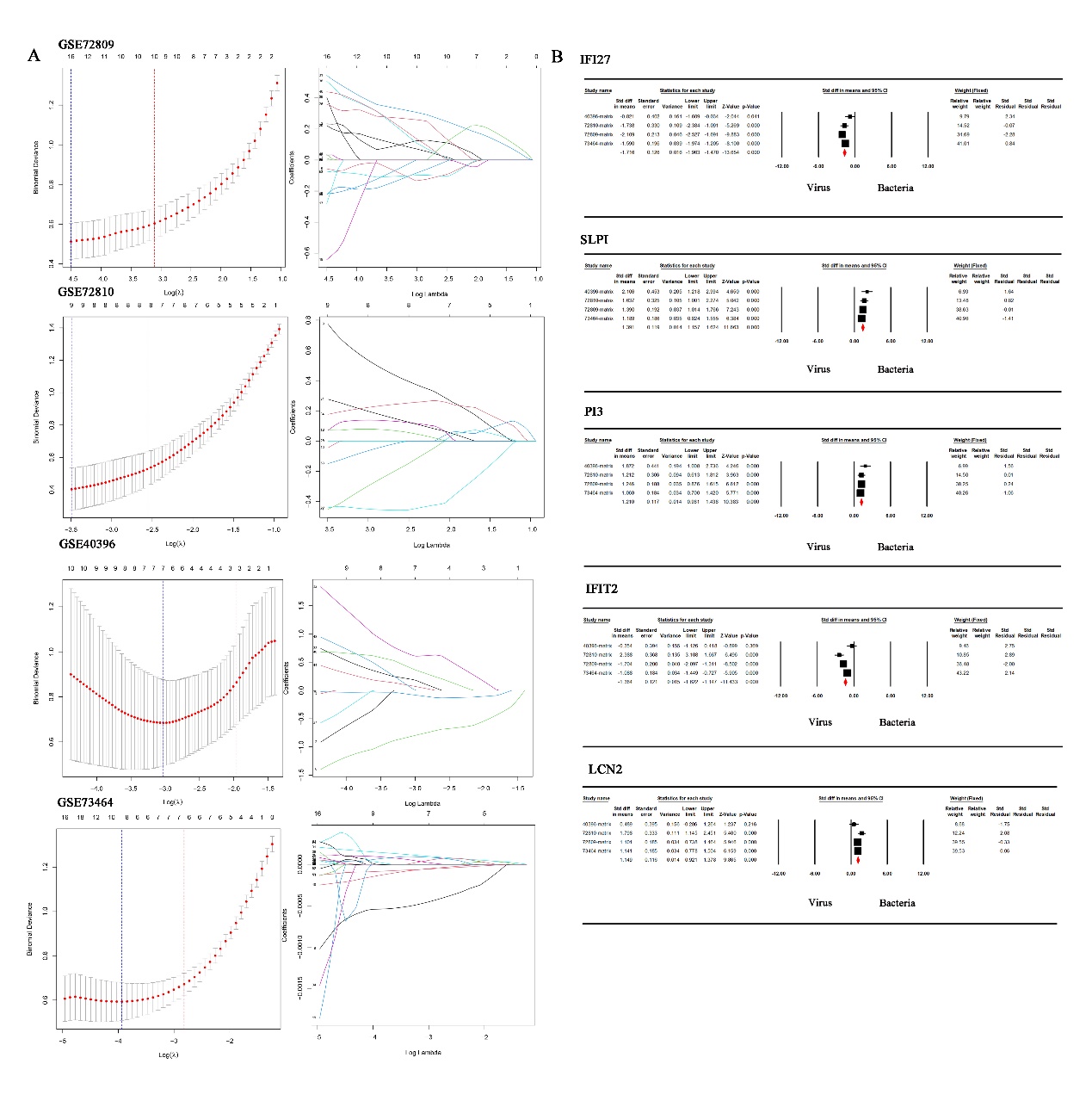


**Supplementary file 7 Immune infiltration analysis in febrile children with B/V infections**

CIBERSORTx is a tool based on the principle of linear support vector regression for the deconvolution analysis of immune cell subtypes(Newman et al., 2019; Steen et al., 2020). In this study, the CIBERSORTx algorithm was used for online analysis to determine the proportional changes of immune cell populations in bacterial or viral febrile illnesses (**Supplementary Fig. 7A-B**). A total of 11 subtypes were identified with a significance criterion of *p<0.05* by CIBERSORTx, including regulatory T cells (Tregs), naive CD4 T cells, activated CD4 memory T cells, resting NK cells, neutrophils, monocytes, M0 macrophages, etc. Among these, 4 subtypes were consistently identified across at least three datasets (**Supplementary Fig. 10**). In addition, the forest plot results indicated that the confidence intervals for macrophages M0 (MD: 0.794, 95% CI: 0.574 to 1.013), resting NK cells (MD: -0.708, 95% CI: -0.926 to -0.491), neutrophils (MD: 0.591, 95% CI: 0.375 to 0.807), and naive CD4+ T cells (MD: -0.517, 95% CI: -0.732 to -0.301) did not cross the line of no effect ( **Supplementary Fig. 7C**). On the other hand, a multivariate nomogram analysis was conducted to predict B/V infection using the proportions of macrophages M0, resting NK cells, neutrophils, and naive CD4 T cells. This suggests that the proportion of these four cell types may be potentially beneficial for the diagnosis of infectious diseases in the future.


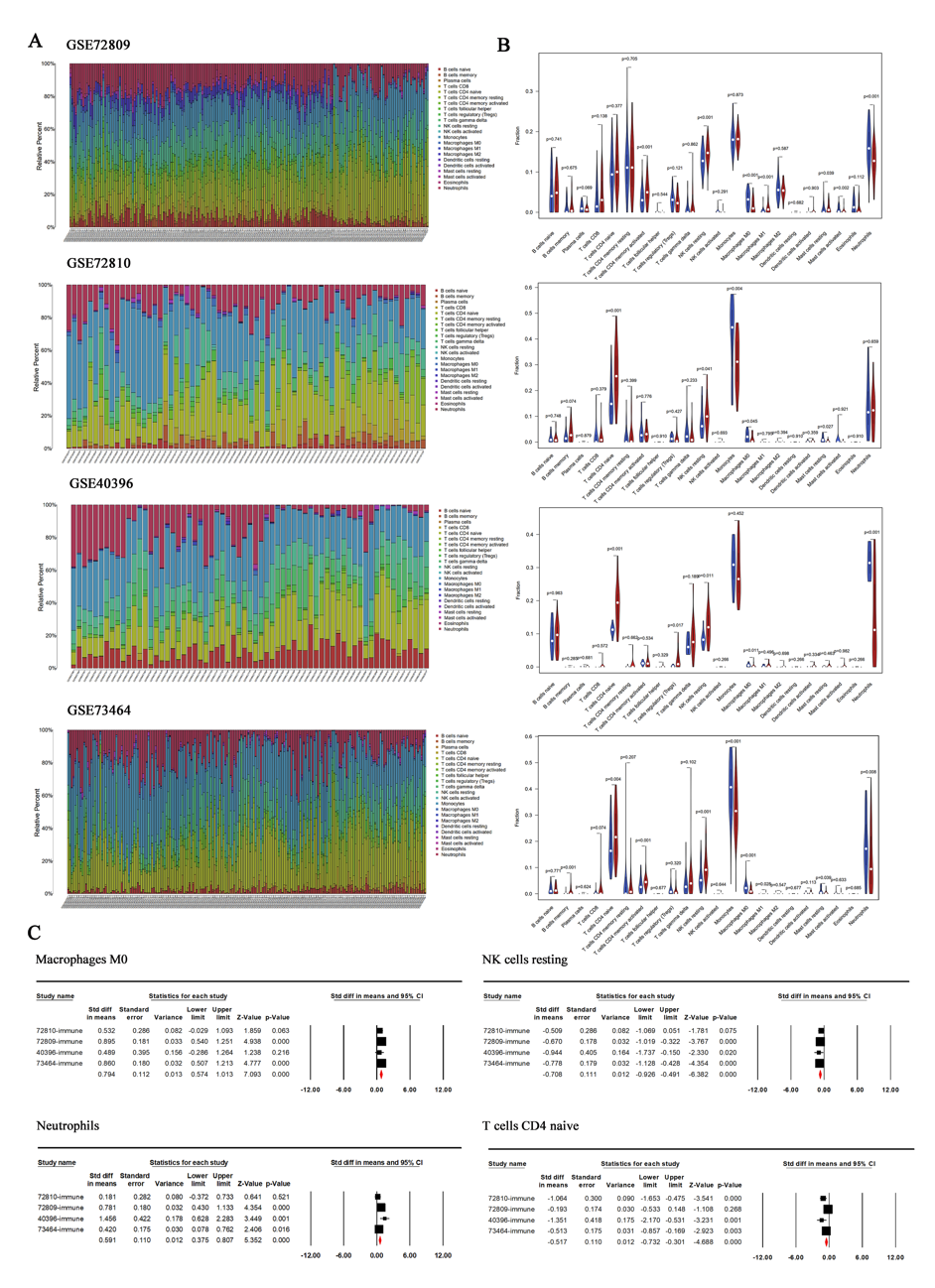


**Supplementary Fig. 7** Gene expression-based proportional immuno-cellular profiling between B/V infections. (A) Differential abundance of immune cell infiltration in 22 types of immune cells was determined for the 384 samples across the datasets GSE40396, GSE72809, GSE72810, and GSE7346. (B) Profiles of immune cell proportions between defined bacterial and viral infections analyzed through CIBERSORTx, with batch correction performed and permutation number setting to100 for significance analysis (Wilcoxon rank sum test). **p<0.05, **p<0.01, ***p<0.001*, ns: no significant. (C) Forest plots of macrophages M0, NK cells resting, neutrophils and T cells CD4 naïve, providing an overview of the confidence intervals for each cell proportion across multiple datasets.

**Supplementary file 8 Nomograms for predicting the type of infection in children with febrile illness.**


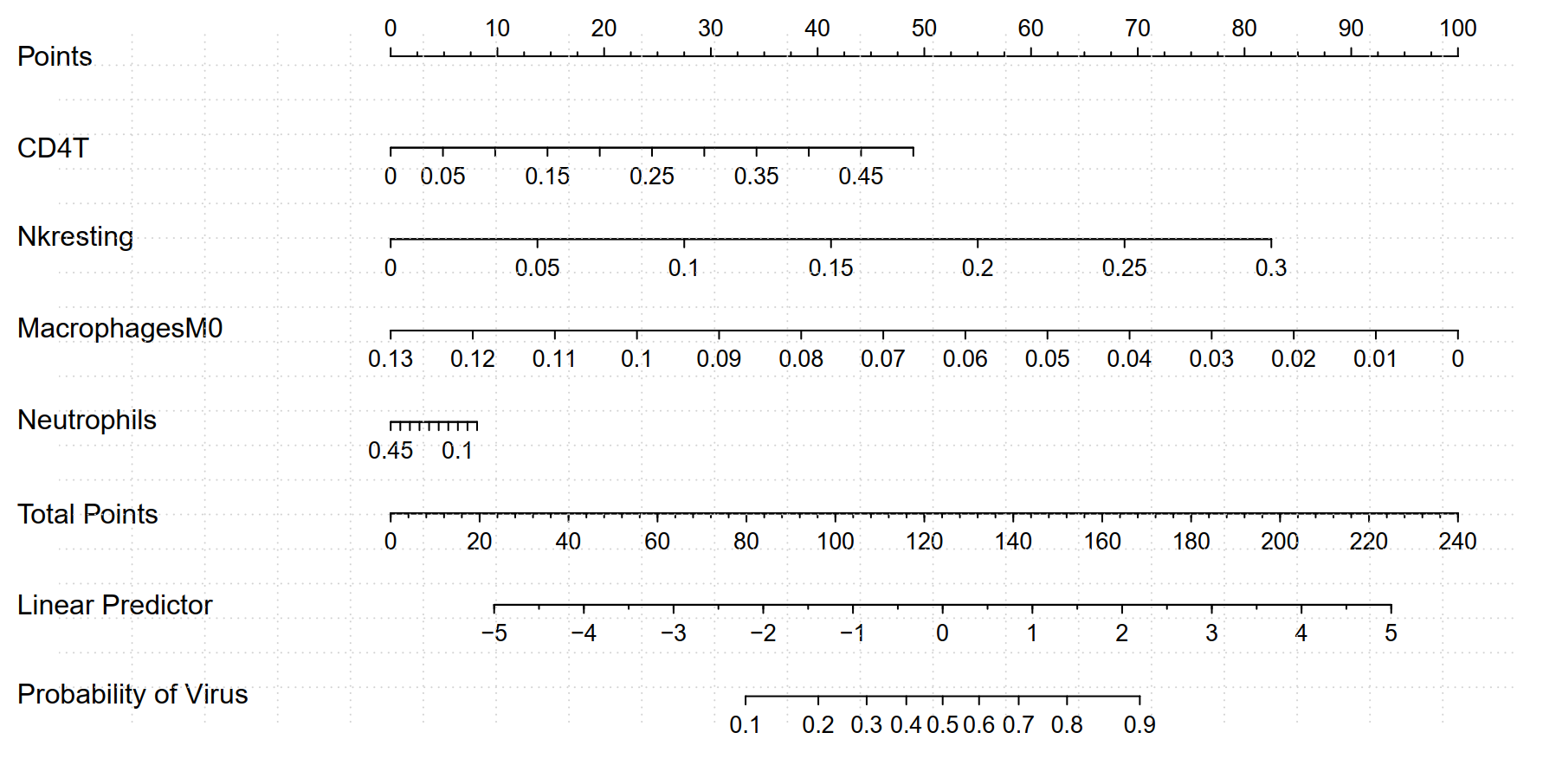


**Supplementary Fig. 8** Nomograms for predicting the type of infection in children with febrile illness. The nomogram analysis examines the variables (M0 macrophages, resting NK cells, neutrophils, and naive CD4 T cells) to predict the probability of B/V infection.

**Supplementary file 9 Table: Information on Five-host-signature genes**

| Gene | Full name | Disease group | Descriptions |
| --- | --- | --- | --- |
| PI3 | Protease Inhibitor 3 | Bacterial | This gene encodes an elastase-specific inhibitor that functions as an antimicrobial peptide against Gram-positive and Gram-negative bacteria, and fungal pathogens. The protein contains a WAP-type four-disulfide core (WFDC) domain, and is thus a member of the WFDC domain family. Most WFDC gene members are localized to chromosome 20q12-q13 in two clusters: centromeric and telomeric. This gene belongs to the centromeric cluster. Expression of this gene is upregulated by bacterial lipopolysaccharides and cytokines. [provided by RefSeq, Oct 2014] |
| IFI27 | Interferon Alpha Inducible Protein 27 | Viral | Enables several functions, including RNA polymerase II-specific DNA-binding transcription factor binding activity; identical protein binding activity; and lamin binding activity. Involved in several processes, including defense response to symbiont; protein K48-linked ubiquitination; and pyroptotic inflammatory response. Acts upstream of or within negative regulation of transcription by RNA polymerase II and regulation of protein export from nucleus. Located in mitochondrial membrane and nuclear inner membrane. Is active in mitochondrion. [provided by Alliance of Genome Resources, Nov 2024] |
| LCN2 | Lipocalin 2 | Viral | This gene encodes a protein that belongs to the lipocalin family. Members of this family transport small hydrophobic molecules such as lipids, steroid hormones and retinoids. The protein encoded by this gene is a neutrophil gelatinase-associated lipocalin and plays a role in innate immunity by limiting bacterial growth as a result of sequestering iron-containing siderophores. The presence of this protein in blood and urine is an early biomarker of acute kidney injury. |
| IFIT2 | Interferon Induced Protein with Tetratricopeptide Repeats 2 | Viral | Enables RNA binding activity. Involved in antiviral innate immune response and positive regulation of apoptotic process. Located in endoplasmic reticulum. [provided by Alliance of Genome Resources, Nov 2024] |
| SLPI | Secretory Leukocyte Peptidase Inhibitor | Bacterial | This gene encodes a secreted inhibitor which protects epithelial tissues from serine proteases. It is found in various secretions including seminal plasma, cervical mucus, and bronchial secretions, and has affinity for trypsin, leukocyte elastase, and cathepsin G. Its inhibitory effect contributes to the immune response by protecting epithelial surfaces from attack by endogenous proteolytic enzymes. This antimicrobial protein has antibacterial, antifungal and antiviral activity |

Note: From NCBI Gene Summary (https://www.ncbi.nlm.nih.gov/gene/?term=)/GeneCards Summary (https://www.genecards.org/)

**Supplementary file 10 Table: The proportional changes of immune cell populations in bacterial or viral febrile illnesses infections.**

| **Access Number** | **Cell** | **P value** |
| --- | --- | --- |
| GSE72810 | T cells CD4 naive | 0.000746 |
|  | NK cells resting | 0.041124 |
|  | Monocytes | 0.003764 |
|  | Macrophages M0 | 0.044503 |
|  | Neutrophils | 0.02665 |
| GSE72809 | T cells CD4 memory activated | 0.000339368 |
|  | NK cells resting | 0.000495199 |
|  | Macrophages M0 | 2.67E-06 |
|  | Macrophages M1 | 3.08E-05 |
|  | Mast cells resting | 0.03903265 |
|  | Mast cells activated | 0.002236132 |
|  | Neutrophils | 2.40E-05 |
| GSE40396 | T cells CD4 naive | 0.000754604 |
|  | T cells regulatory (Tregs) | 0.017210933 |
|  | NK cells resting | 0.011016222 |
|  | Macrophages M0 | 0.010803135 |
|  | Neutrophils | 0.000754604 |
| GSE73464 | B cells memory | 0.00039175 |
|  | T cells CD4 naive | 0.003886735 |
|  | T cells CD4 memory activated | 0.000268622 |
|  | NK cells resting | 9.89E-07 |
|  | Monocytes | 0.00010611 |
|  | Macrophages M0 | 3.30E-06 |
|  | Macrophages M1 | 0.028300729 |
|  | Mast cells resting | 0.038536518 |
|  | Neutrophils | 0.008091399 |
|  |  |  |

**Supplementary file 11 The input data format for rf_app.R**

The input file for the R application (rf_app.R) (.CSV)

𝑅𝑒𝑓𝑉𝑎𝑙𝑢𝑒(𝑖) = 𝑆𝑖𝑔𝑚𝑜𝑖𝑑 [𝑒𝑥𝑝𝑟. 𝑣𝑎𝑙𝑢𝑒 (𝑖) /𝑒𝑥𝑝𝑟. 𝑣𝑎𝑙𝑢𝑒 (ℎ𝑜𝑢𝑠𝑒𝑘𝑒𝑒𝑝𝑖𝑛𝑔 𝑔𝑒𝑛𝑒 RPLP0)].

𝑅𝑒𝑓𝑉𝑎𝑙𝑢𝑒 (IFI27, LCN2, IFIT2, PI3, SLPI) were input values.

Id IFI27 LCN2 IFIT2 PI3 SLPI Probable Status

1 x x x x x Bacterial/Viral

2 x x x x x Bacterial/Viral

3 x x x x x Bacterial/Viral

**Supplementary file 12 Artificial neural network output R code**

<?xml version="1.0" encoding="UTF-8" ?>

<PMML

version="4.0"

xmlns="http://www.dmg.org/PMML-4_0"

xmlns:xsi="http://www.w3.org/2001/XMLSchema-instance"

xsi:schemaLocation="http://www.dmg.org/PMML-4_0 pmml-4-0.xsd">

<Header

copyright="Copyright (c) IBM Corp. 1999, 2011.">

<Application

name="IBM SPSS Statistics"

version="20.0.0.0"/>

<Timestamp>Thu Dec 19 15:40:30 2024</Timestamp>

</Header>

<DataDictionary

numberOfFields="6">

<DataField

name="status"

optype="categorical"

dataType="double">

<Value

value="0"

displayValue="Bacterial"/>

<Value

value="1"

displayValue="Viral"/>

</DataField>

<DataField

name="IFI27"

optype="continuous"

dataType="double"/>

<DataField

name="LCN2"

optype="continuous"

dataType="double"/>

<DataField

name="IFIT2"

optype="continuous"

dataType="double"/>

<DataField

name="PI3"

optype="continuous"

dataType="double"/>

<DataField

name="SLPI"

optype="continuous"

dataType="double"/>

</DataDictionary>

<TransformationDictionary>

<DerivedField

optype="categorical"

dataType="double"

name="statusValue0">

<NormDiscrete

field="status"

value="0"/>

</DerivedField>

<DerivedField

optype="categorical"

dataType="double"

name="statusValue1">

<NormDiscrete

field="status"

value="1"/>

</DerivedField>

<DerivedField

optype="continuous"

dataType="double"

name="IFI27Norm">

<NormContinuous

field="IFI27">

<LinearNorm

orig="0.500058168"

norm="-1.89791414081241"/>

<LinearNorm

orig="0.999997683"

norm="2.12374516372781"/>

</NormContinuous>

</DerivedField>

<DerivedField

optype="continuous"

dataType="double"

name="LCN2Norm">

<NormContinuous

field="LCN2">

<LinearNorm

orig="0.503731099"

norm="-1.63795090720186"/>

<LinearNorm

orig="0.892870744"

norm="1.8130993232388"/>

</NormContinuous>

</DerivedField>

<DerivedField

optype="continuous"

dataType="double"

name="IFIT2Norm">

<NormContinuous

field="IFIT2">

<LinearNorm

orig="0.502332202"

norm="-1.83302991900428"/>

<LinearNorm

orig="0.999738487"

norm="2.55236531936416"/>

</NormContinuous>

</DerivedField>

<DerivedField

optype="continuous"

dataType="double"

name="PI3Norm">

<NormContinuous

field="PI3">

<LinearNorm

orig="0.499505567"

norm="-1.3222963999388"/>

<LinearNorm

orig="0.907413673"

norm="2.48078108809498"/>

</NormContinuous>

</DerivedField>

<DerivedField

optype="continuous"

dataType="double"

name="SLPINorm">

<NormContinuous

field="SLPI">

<LinearNorm

orig="0.499952647"

norm="-1.23214296936696"/>

<LinearNorm

orig="0.812936486"

norm="1.94348147092597"/>

</NormContinuous>

</DerivedField>

</TransformationDictionary>

<NeuralNetwork

functionName="classification"

activationFunction="tanh">

<MiningSchema>

<MiningField

name="IFI27"/>

<MiningField

name="LCN2"/>

<MiningField

name="IFIT2"/>

<MiningField

name="PI3"/>

<MiningField

name="SLPI"/>

<MiningField

name="status"

usageType="predicted"/>

</MiningSchema>

<NeuralInputs>

<NeuralInput

id="0">

<DerivedField

optype="continuous"

dataType="double">

<FieldRef

field="IFI27Norm"/>

</DerivedField>

</NeuralInput>

<NeuralInput

id="1">

<DerivedField

optype="continuous"

dataType="double">

<FieldRef

field="LCN2Norm"/>

</DerivedField>

</NeuralInput>

<NeuralInput

id="2">

<DerivedField

optype="continuous"

dataType="double">

<FieldRef

field="IFIT2Norm"/>

</DerivedField>

</NeuralInput>

<NeuralInput

id="3">

<DerivedField

optype="continuous"

dataType="double">

<FieldRef

field="PI3Norm"/>

</DerivedField>

</NeuralInput>

<NeuralInput

id="4">

<DerivedField

optype="continuous"

dataType="double">

<FieldRef

field="SLPINorm"/>

</DerivedField>

</NeuralInput>

</NeuralInputs>

<NeuralLayer

numberOfNeurons="4">

<Neuron

id="5"

bias="-0.294017051138455">

<Con

from="0"

weight="0.788216989233615"/>

<Con

from="1"

weight="-0.869623017727042"/>

<Con

from="2"

weight="0.331566103487412"/>

<Con

from="3"

weight="-0.535217299928165"/>

<Con

from="4"

weight="-0.263439038789156"/>

</Neuron>

<Neuron

id="6"

bias="-0.342950229787619">

<Con

from="0"

weight="-0.685499090125939"/>

<Con

from="1"

weight="1.43588812857516"/>

<Con

from="2"

weight="-0.513940329520469"/>

<Con

from="3"

weight="1.35086645324322"/>

<Con

from="4"

weight="1.04265395787008"/>

</Neuron>

<Neuron

id="7"

bias="-0.579546513202033">

<Con

from="0"

weight="-0.528383961248397"/>

<Con

from="1"

weight="0.652765340458114"/>

<Con

from="2"

weight="-0.849364112683968"/>

<Con

from="3"

weight="0.0943670377978737"/>

<Con

from="4"

weight="0.331512667480932"/>

</Neuron>

<Neuron

id="8"

bias="-0.0384012174258297">

<Con

from="0"

weight="-0.597496686290152"/>

<Con

from="1"

weight="0.293420621829429"/>

<Con

from="2"

weight="-0.543220151981133"/>

<Con

from="3"

weight="0.408542902856375"/>

<Con

from="4"

weight="0.304317267582946"/>

</Neuron>

</NeuralLayer>

<NeuralLayer

numberOfNeurons="3">

<Neuron

id="9"

bias="-0.354658042585451">

<Con

from="5"

weight="0.0929156706799415"/>

<Con

from="6"

weight="0.617428321850961"/>

<Con

from="7"

weight="0.763763101952283"/>

<Con

from="8"

weight="0.749868940833202"/>

</Neuron>

<Neuron

id="10"

bias="0.728614387997229">

<Con

from="5"

weight="0.00795689675504007"/>

<Con

from="6"

weight="-0.242233413785548"/>

<Con

from="7"

weight="0.416432455447462"/>

<Con

from="8"

weight="0.411878277800174"/>

</Neuron>

<Neuron

id="11"

bias="0.334862813658474">

<Con

from="5"

weight="-0.766653988503901"/>

<Con

from="6"

weight="-0.414130982675169"/>

<Con

from="7"

weight="-1.52362744845771"/>

<Con

from="8"

weight="-0.673258887131744"/>

</Neuron>

</NeuralLayer>

<NeuralLayer

numberOfNeurons="2"

activationFunction="identity"

normalizationMethod="softmax">

<Neuron

id="12"

bias="-0.175730416054074">

<Con

from="9"

weight="0.900234558815856"/>

<Con

from="10"

weight="0.52160623188528"/>

<Con

from="11"

weight="-0.774892314117695"/>

</Neuron>

<Neuron

id="13"

bias="0.210189097063785">

<Con

from="9"

weight="-1.53800162273531"/>

<Con

from="10"

weight="-1.26177383524763"/>

<Con

from="11"

weight="0.521257338360245"/>

</Neuron>

</NeuralLayer>

<NeuralOutputs>

<NeuralOutput

outputNeuron="12">

<DerivedField

optype="categorical"

dataType="double">

<FieldRef

field="statusValue0"/>

</DerivedField>

</NeuralOutput>

<NeuralOutput

outputNeuron="13">

<DerivedField

optype="categorical"

dataType="double">

<FieldRef

field="statusValue1"/>

</DerivedField>

</NeuralOutput>

</NeuralOutputs>

</NeuralNetwork>

</PMML>
